# Educational standards for Australian social prescribing link workers: a modified Delphi study

**DOI:** 10.3389/fpubh.2026.1754668

**Published:** 2026-02-19

**Authors:** J. R. Baker, Alessandra K. Teunisse, Yvonne Zurynski, Christina Aggar, Thomas Astell-Burt, Xiaoqi Feng, Michelle Bissett, Rosanne Freak-Poli

**Affiliations:** 1 Faculty of Health, Southern Cross University, Bilinga, QLD, Australia; 2 Primary & Community Care Services Limited, Thornleigh, NSW, Australia; 3 Australian Social Prescribing Institute of Research and Education, Surry Hills, NSW, Australia; 4 Australian Institute of Health Innovation, Macquarie University, Sydney, NSW, Australia; 5 School of Architecture, Design and Planning, University of Sydney, Sydney, NSW, Australia; 6 Westmead Applied Research Centre, Westmead Hospital, Sydney, NSW, Australia; 7 Population Wellbeing and Environment Research Lab (PowerLab), Sydney, NSW, Australia; 8 Charles Perkins Centre, University of Sydney, Sydney, NSW, Australia; 9 Sydney Environment Institute, University of Sydney, Sydney, NSW, Australia; 10 School of Population Health, University of New South Wales, Sydney, NSW, Australia; 11 The George Institute for Global Health, Sydney, NSW, Australia; 12 Chronic Disease and Ageing, School of Public Health and Preventative Medicine, Monash University, Melbourne, VIC, Australia; 13 Medicine Monash Health, School of Clinical Sciences, Monash University, Melbourne, VIC, Australia

**Keywords:** Australia, competencies, Delphi technique, educational standards, graduate attributes, healthcare workforce, link worker, social prescribing

## Abstract

**Background:**

Social prescribing integrates comprehensive health and wellbeing outcomes into routine medical care, addressing social determinants alongside biomedical treatment to support prevention and quality of life. In Australia, social prescribing is increasingly recognised in policy with the optimal model involving link workers delivering the intervention. However, no standardised educational framework exists for this emerging workforce. We aimed to develop consensus-based national education standards for social prescribing link workers through stakeholder consultation.

**Methods:**

A modified Delphi technique evaluated draft graduate attributes and core competencies through online surveys, with experts rating agreement on 5-point Likert scales. Consensus was defined as ≥80% agreement without critical revision needed as identified in qualitative feedback. Participants included Australian and international experts representing healthcare providers, academics, researchers, educators, and community organisations, recruited through purposive sampling. International experts provided benchmarking against established frameworks. Qualitative data informed revisions between rounds.

**Results:**

Round 1 involved 36 Australian and 11 international experts. Consensus was achieved on seven graduate attributes (Ethical Practitioner, Inclusive and Respectful, Effective Communicator, Critical Thinker and Problem Solver, Systemic Change Catalyst, Community Empowerment and Collaboration, Reflective and Lifelong Learner) and nine core competencies (spanning Australian health system navigation, community development, cultural safety, and evaluation). Round 2 (33 Australian, 10 international) achieved consensus on all revised items.

**Conclusion:**

These standards provide the first consensus-based framework for Australian social prescribing link worker education addressing unique contextual requirements while balancing comprehensive capabilities with practical implementation. The standards represent expert agreement on educational priorities rather than empirically validated outcomes, and require testing through implementation in training programs and practice settings, alongside planned qualitative research with community stakeholders.

## 1 Introduction

The World Health Organization (WHO) recently declared that “social connection is essential for the health, strength and resilience of individuals and societies” (1) and identified the health sector as having a central role (1). Australia faces mounting healthcare challenges that demand innovative workforce solutions. Rising rates of chronic and developmental conditions among adolescents (with nearly half now living with conditions such as ADHD, asthma, or autism spectrum disorder), increasing mental health disorders in young adults (from 26 to 39% between 2007 and 2021), and an ageing population expected to double in coming decades are placing unprecedented pressure on clinical services (2–4). Simultaneously, health professional shortages across general practice, nursing, and mental health represent one of the most undersupplied workforce areas (5). These converging trends underscore the urgent need for scalable, non-clinical interventions that can address social determinants of health while supporting overstretched clinical systems.

Social prescribing, an evidence-based mechanism that complements biomedical treatment, is commonly defined as a process whereby trusted individuals in clinical and community settings identify non-medical, health-related social needs and subsequently connect people to non-clinical supports and services within their communities (6). A distinguishing feature of social prescribing is the collaborative process of co-designing personalised plans with social prescriptions (7). The personalised plan can include accessing multiple community-based interventions including practical assistance with material needs (e.g., food, housing, transportation), skill development and educational opportunities (e.g., volunteering, job training, adult education), and activities that promote social connection and community engagement (e.g., befriending, arts, physical activity). This approach directly responds to healthcare capacity constraints by enabling non-clinical practitioners to address the psychosocial factors that often underlie or exacerbate health conditions, potentially preventing escalation to more intensive clinical interventions. Ideally, social prescribing enables a broad range of practical, social and structural determinants of health to be addressed through tailored, individualised solutions that align with participants’ specific needs and interests by leveraging existing health, societal, and community systems, social prescribing can potentially address the psychosocial factors that may affect or exacerbate health conditions (8). Many international models of social prescribing, such as Canadian (9) and British models (10), also include aspects of community building and adapting interventions to local contexts (11).

The effectiveness of social prescribing is well-documented, with research demonstrating that social prescribing can reduce anxiety, depression, social isolation, and loneliness, and improve mental wellbeing, (12–18) as well as improve self-reported health (18–21), general wellbeing (19, 20), quality of life (13, 18, 21, 22), and self-esteem (12, 20). Central to many successful social prescribing initiatives is the link worker, also known as a connector, navigator, or social prescriber (23). Social prescribing typically operates through two main models: the direct referral model and the link worker model. In the direct referral model, healthcare professionals [e.g., general practitioners (GP) or nurses] or trusted people (e.g., teachers) refer patients directly to local programs or services. While this approach may appear straightforward, it is actually resource-intensive as it requires highly paid healthcare professionals to spend considerable time on non-clinical activities, creating opportunity costs and workforce strains as these referrers work outside their core scope of practice and lack the specialised community knowledge needed for effective social prescribing (24). By contrast, the link worker model inserts a trained intermediary—commonly referred to as a link worker—between the initial referrer (whether clinician, teacher, or other trusted professional and community resources). Link workers, according to the World Health Organization, are typically community-based professionals or trained volunteers who possess good understanding of their communities and ideally have experience supporting people. They may be existing staff (such as social workers, community health workers, or nurses) taking on expanded roles, or newly recruited positions designed specifically for this purpose (24). Link workers engage in collaborative conversations with referred individuals, identifying needs, preferences, and goals to co-develop a personalised plan and facilitate connections to suitable services. The link worker model offers several advantages: link workers typically possess in-depth knowledge of local resources; they provide continuity through follow-up and monitoring; and they reduce the burden on GPs and other referrers by addressing non-medical issues that would otherwise return repeatedly to clinical settings. These factors not only improve health and wellbeing outcomes but also create system efficiencies through reduced primary care consultations, emergency department attendances, and secondary care referrals, with studies showing evidence of long-term cost savings (24, 54).

Link workers have been shown to build confidence, motivation, knowledge, and the skills needed to support and improve wellbeing (25). A review of social prescribing studies found that knowledgeable and well-trained link workers are beneficial and necessary to the process but not sufficient due to the many interacting elements on the referral pathways (26). A growing body of international research highlights that successful social prescribing depends on link workers who combine practical knowledge with multifaceted social and emotional skills, and who can build trust-based, supportive relationships over time (27–30). Additionally, Wildman et al. (31) found that a strong, supportive relationship with an accessible link worker was crucial for sustaining beneficial behaviours, particularly for individuals facing complex health and socioeconomic challenges who often require longer-term intervention and personalised support. This relationship typically involves more frequent contact initially to build trust and momentum, particularly important given that many clients may have experienced social isolation, mental health challenges, or previous negative experiences with services, before gradually reducing contact as individuals become more empowered and self-directed.

While link worker models have been successfully implemented internationally, particularly in the United Kingdom’s NHS and Canada’s community health networks, Australia’s healthcare context presents distinct challenges that necessitate locally adapted standards. Unlike single-payer systems such as England’s NHS, Australia operates within a complex multi-funder environment incorporating Medicare, the National Disability Insurance Scheme (NDIS), My Aged Care, and state-based health services, requiring link workers to navigate multiple funding streams and eligibility criteria. Australia’s vast geography, ranging from metropolitan centres to remote communities, combined with the world’s oldest continuous living culture among First Nations peoples (32), creates unique implementation requirements not adequately addressed by existing international frameworks.

Internationally, social prescribing is at varying stages of adoption. In Australia, it is increasingly recognised in policy as a tool to strengthen prevention, primary care, and social connection (33, 34). To drive implementation, the Australian Social Prescribing Institute of Research and Education (ASPIRE) was established in 2019 to provide leadership through research, advocacy, and development of co-designed frameworks tailored to the Australian health system structure and demographic and cultural contexts (35). In 2024, ASPIRE convened over 50 national organisations at the *Accelerating Social Prescribing Roundtable* in Canberra, co-producing a Consensus Statement outlining key principles and priorities for national development and implementation (36). Workforce development was identified as one of the critical priorities, with the Consensus Statement specifically calling for comprehensive education, training and professional networking support for both referrers and link workers to ensure effective service delivery.

While the global and national momentum is clear, a critical challenge remains: the health workforce must be adequately equipped to deliver social prescribing effectively. This includes both upskilling the existing health workforce, especially workforce providing primary care, to identify and refer individuals in need and establishing a dedicated workforce of trained link workers. However, awareness and understanding of social prescribing and link worker roles remains limited among many healthcare professionals, with studies indicating that frontline clinicians often lack knowledge of what social prescribing involves or how link workers function (28, 29). Furthermore, standards for link workers are underdeveloped and inconsistent across jurisdictions. Without such standards, link workers face variable role expectations, inadequate training, and limited support, which in turn compromises their confidence, professional identity, and capacity to deliver equitable and high-quality care.

Developing national education standards for social prescribing link workers is essential for several reasons. First, standards would ensure consistency in education and training across diverse health settings and jurisdictions, which should then lead to more standardised care delivery and prevent inequities in service delivery among regions or populations and ensuring equitable access to high-quality support regardless of geography. Second, they would build the confidence and professional credibility of link workers, enabling clearer role boundaries, strengthening professional recognition, and facilitating integration within primary care and community health teams. Third, educational standards would identify what knowledge, skills and values are essential and then guide training and professional development pathways, ensuring link workers possess the identified and necessary competencies. Finally, establishing standards is critical for sustainability: providing funders, commissioners, and policymakers with assurance that link worker services are evidence-based, accountable, and cost-effective, while offering a framework for accountability, cost-effectiveness, and impact evaluation that gives governments the confidence to embed social prescribing in long-term preventive health strategies. These standards could also inform the direct referral model; however we acknowledge the time commitment and financial cost required for training is likely a barrier for health professionals and trusted people in the community.

The aim of this study was to develop consensus-based national education standards for social prescribing link workers in Australia. To achieve this aim, we undertook a consensus-based approach through a Delphi study, integrating perspectives from a broad range of stakeholders, including academic researchers, not-for-profit and community organisations, industry partners, current link workers, and representatives from government and policy.

## 2 Method

### 2.1 Study design

The Delphi technique was selected as the most appropriate methodology for developing these educational standards due to its established effectiveness in achieving expert consensus through structured, iterative discussions (37, 38). The Delphi method is particularly valuable when developing professional standards or guidelines that require both theoretical rigour and practical applicability (39). This approach provided a structured “ground-up” approach to gaining reliable expert consensus, ensuring that the resulting standards would reflect both academic expertise and practical implementation experience. The methodology also aligned well with our aim to develop standards that would be responsive to Australia’s unique health system characteristics and geographical diversity. By engaging participants (defined in section 3.3) from various regions and professional backgrounds, the Delphi process facilitated the development of standards that could be implemented effectively across different healthcare settings while maintaining consistency in quality and outcomes. As well as using a large group of Australian participants, a small panel of international participants were also sampled to benchmark the standards globally. This study was conducted and reported with consideration of both the Conducting and Reporting Delphi Studies guidelines (40) and the reporting guidelines for Delphi techniques in health sciences (41). Alternative methodologies were considered. Consensus development conferences or nominal group techniques would have required synchronous participation, which was impractical given the geographic dispersion of Australian experts and the inclusion of international participants for benchmarking purposes. Mixed methods approaches combining surveys with focus groups were considered, but the Delphi’s structured iterative process was deemed more appropriate for achieving systematic consensus across a large, geographically dispersed expert panel while maintaining anonymity to reduce dominance effects and social pressure that can occur in face-to-face consensus methods.

### 2.2 Educational standards for Australian social prescribing link workers

The draft educational standards that formed the basis for this Delphi study were developed through an iterative synthesis process appropriate for an emerging field with limited published frameworks on link worker education. The development combined three evidence sources: (1) the lead author and colleague’s ongoing engagement with peer-reviewed and grey literature on social prescribing and link worker roles from Australia and internationally, including publications examining various aspects of social prescribing in Australia (17, 21, 22, 42–46) and international practices from statutory entities such as the World Health Organization and the NHS; (2) consultations with implementation leaders across six countries (Canada, Netherlands, UK, Singapore, Austria, and Poland) exploring their local link worker models, training elements, and contextual adaptations; and (3) over a decade of practical experience implementing and supervising social prescribing programs across diverse Australian contexts.

To mitigate potential bias from practice-based insights, the draft framework prioritised competencies that appeared across multiple evidence sources and aligned with documented implementation experiences. This preliminary framework was then subjected to systematic expert consensus through the Delphi process described below. The development of the draft training standards considered the Australian context to ensure relevance. In Australia, social prescribing operates within a complex healthcare landscape characterised by multiple funding streams, diverse geographical contexts, and rich cultural diversity, which includes the largest living first nations culture (32, 47–50). This complexity creates distinct challenges for implementing social prescribing effectively. The draft standards comprise two main components: graduate attributes and core competencies (Supplementary Table 1). The graduate attributes outline key characteristics expected of social prescribing Link Workers upon completion of their training while the core competencies detail the specific abilities required for effective practice in the Australian context.

### 2.3 Participants

Participants were recruited using purposive sampling, with expertise defined as: ASPIRE membership; authorship of academic or grey literature on social prescribing; research involvement in social prescribing; health or social care provision involving social prescribing; Link Worker experience; or healthcare administration/management of social prescribing services. Given the emerging nature of the field, participants were included even when their work was not explicitly labelled as ‘social prescribing’ or ‘link work’. Diversity in professions was prioritised to mitigate professional biases in cross-disciplinary Delphi studies with heterogeneous samples. Participants were included if they understood the Australian social prescribing landscape and able to read and understand English.

Sample size considerations for expert panels in a Delphi study varies, with the average sample size in the health sciences being 40 participants (39) and most ranging from 20 to 60 (6), while specialised topics may only require15-20 (51). Accounting for the expected dropout rate of 20–30% between subsequent rounds (51), we aimed to recruit 40 Australian experts, with an additional subgroup of 10 international experts for benchmarking.

### 2.4 Recruitment

Potential participants were identified through networks developed during previous studies on social prescribing, contacts known to the research team, relevant published research and news articles, attendees at social prescribing conferences, and member directories of professional associations relevant to social prescribing. Invitations to participate were emailed to potential participants, along with a detailed information sheet, contact details of the researchers, and the link to Round 1 of the study. Additional invitations were sent to a purposive selection of ‘snowballed’ contacts whose professional expertise was not yet adequately represented in the sample to ensure diversity.

To enhance representativeness within the constraints of an emerging field, several strategies were employed. First, we systematically sought participants across multiple expertise types (providers, researchers, policy makers, consumers) and geographical regions within Australia to capture diverse implementation contexts. Second, the snowball sampling approach specifically targeted gaps in representation identified during initial recruitment, such as ensuring adequate representation from rural/regional areas, diverse organisational types (government, NGO, private), and practitioners with direct link worker experience. Third, international expert inclusion provided external benchmarking perspectives. However, we acknowledge that true population-level representativeness was not feasible given the nascent state of social prescribing in Australia, where the total population of individuals with relevant expertise is unknown and formal professional registration for link workers does not yet exist. Therefore, our sampling aimed for diversity of expertise and perspective rather than statistical representativeness.

### 2.5 Data collection

The Delphi studies were conducted over 4 months using the Qualtrics™ survey management platform. Data were collected using unique survey links to enable follow-up while maintaining participant confidentiality. Participants provided informed consent electronically before accessing the survey content. Unique survey links were emailed to maintain participant confidentiality while enabling follow-up communications. Two follow-up reminder emails were sent at one-week intervals to non-responders.

#### 2.5.1 Round one

The first round was conducted between 23rd of October and 6th of November 2024. Participants completed a 45-min survey containing 143 items across four sections: participant demographics (8 questions), graduate attributes (7 questions) and key indicators of the graduate attribute (37 questions), core competencies (9 questions) and associated skills with each core competency (63 questions), and qualitative feedback (19 questions). The graduate attributes and core competencies were derived from the draft educational standards developed through synthesis of existing evidence, international benchmarking, and practical implementation experience (see section 3.2) Demographics covered age, gender, indigenous status, location, expertise, experience, education, and organisational affiliation.

For each of the seven graduate attributes and nine core competencies, participants rated their agreement on a 5-point Likert scale from 1 (*Completely Disagree*) to 5 (*Completely Agree*). For graduate attributes, participants evaluated both the overall attribute and its specific indicators, with questions asking if they agreed these were key elements for the Link Worker role. Similarly, for core competencies, participants rated both the overall competency and its associated skills, as detailed in the initial draft framework. Open-text fields followed each section for additional comments. The final question of the survey was “*Is there anything else you would like to comment on regarding these educational standards for Social Prescribing Link Workers in Australia?*”

#### 2.5.2 Round two

The second round was conducted between 25th of November 2024 and 7th of February 2025. This round had a total of 25 questions and took an estimated 15 min to complete: 6 questions for the revised graduate attributes, 10 questions for the revised core competencies, 8 questions for new skills added to existing competencies, and an opportunity for final comments. In the revised graduate attributes section, 3 new indicators were developed based on participant feedback to replace ones that did not meet consensus. For each item, participants were asked “*Please rate your overall level of agreement with this revision*” on a 5-point Likert-style scale from 1 (*Completely Disagree*) to 5 (*Completely Agree*), as well as “*Do you have any comments on this?*.” In the revised core competencies section, 5 revised skills were developed based on Round 1 feedback to replace ones that did not meet consensus, and 4 new skills emerged from thematic analysis of qualitative responses. The same two questions were asked for those revised and new skills. The final question was “*Thinking about these educational standards as a whole, do you have any final comments on the social prescribing link worker framework?*”

#### 2.5.3 Participant burden

Respondent burden was mitigated through several design features. The survey structure prioritised quantitative ratings over written responses, with qualitative feedback fields optional for each section. This allowed participants to provide detailed commentary where they felt it valuable whilst minimising time demands. Round 2 was substantially shorter than Round 1 (15 min vs. 45 min), focusing only on items requiring revision. The timeframe between rounds was kept brief to maintain participant engagement whilst allowing sufficient time for analysis and revision. Within-survey completion rates were high, with 86% of Australian participants in Round 1 completing all questions (31 of 36 who started). All Round 2 participants (Australian *n* = 33, International *n* = 10) completed the quantitative items, though three international participants did not respond to the final three questions. Qualitative response rates varied, with 74% of Round 1 participants and 63% of Round 2 participants providing at least one written comment, suggesting participants selectively provided detailed feedback where they had substantive input rather than feeling obligated to respond to every optional field.

### 2.6 Consensus and data analysis

Although there is not much agreement on what constitutes consensus in the literature (39, 51), there is a suggestion that for the development of medical guidelines, consensus is 75% agreement, and a high level of consensus is 95% agreement (41). For this study, consensus was defined *a priori* if ≥80% of participants rated the item as *Completely Agree* or *Somewhat Agree* combined. Neutral responses were included in the denominator but not counted toward the consensus threshold, treating neutrality as absence of agreement rather than disagreement. This conservative approach ensures that consensus reflects not only lack of disagreement but also sufficient clarity for experts to take definitive supportive positions. Stopping criteria were not predetermined by number of rounds; rather, the decision to conduct additional rounds was guided by whether consensus thresholds were achieved and whether qualitative feedback patterns indicated stable consensus or continued conceptual concerns requiring revision. Previous social prescribing Delphi studies have demonstrated that items achieving 78–79.99% agreement (within 2% of threshold) may indicate practical consensus (6). Items not reaching the 80% threshold were evaluated for whether qualitative feedback indicated fundamental conceptual concerns requiring revision or focused primarily on implementation considerations suggesting practical consensus had been achieved. Data was analysed in Microsoft Excel. Quantitative data were expressed as percentages and qualitative data were analysed using Braun and Clarke’s (52) thematic analysis methodology. This approach was selected at it provided a rigorous and systematic approach to identifying patterns in qualitative data and is well suited to examining different perspectives and highlighting differences and similarities.

The thematic analysis followed Braun and Clarke’s six-phase approach. The second author (AT) led the qualitative analysis with oversight from the first author (JB). Phase 1 (familiarisation) involved multiple readings of all qualitative responses from both Australian (*n* = 36) and International (*n* = 11) participants, with documentation of initial impressions. Phase 2 (initial coding) involved line-by-line coding focusing on explicit and implicit statements about requirements, challenges, role boundaries, scope, and implementation considerations. Phase 3 (theme development) grouped codes into potential themes, creating initial thematic maps and identifying relationships between codes. Phase 4 (theme review) checked themes against original data to ensure they captured the breadth of responses and were coherent and distinct, with cross-checking against quantitative agreement levels to understand patterns of consensus and dissent. Phase 5 (theme definition) developed clear definitions for each theme and selected representative quotes. Phase 6 (reporting) organised themes to address feedback on the standards and broader implementation requirements. The analysis used a combination of inductive (data-driven) and deductive (theory-driven) coding approaches, maintaining distinction between Australian and International responses throughout. Credibility was enhanced through regular discussion between analysts (AT and JB) to review emerging themes, resolve any interpretive differences, and ensure themes accurately reflected participant perspectives. The integration of qualitative and quantitative data ensured that revisions between rounds were informed by both consensus levels and the substantive concerns expressed in open-text responses.

### 2.7 Ethics

This study was reviewed and approved by the human research ethics committee from Monash University (approval no. 44727).

## 3 Results

### 3.1 Participant characteristics

The initial email was sent to 96 experts. In total, 47 completed round 1 (49% response rate). Participants represented diverse expertise types, including healthcare providers/practitioners (*n* = 15), researchers/academics (*n* = 11), government and policy representatives (*n* = 7), and consumers/service users (*n* = 3), with some participants holding multiple roles. Of the 47 participants in Round 1, 43 provided complete responses for round 2 (91% completion rate among those who started the survey) (Table 1). Australian experts had a mean age of 30.3 years (SD = 11.8), were 78% female (*N* = 28), and represented diverse professional backgrounds including health care providers, researchers, educators, and social prescribing practitioners. Most participants were from Queensland (44%, *N* = 16), New South Wales (25%, *N* = 9), and Victoria (22%, *N* = 8), with primary affiliations to non-government organisations, and averaged 17.2 years of relevant expertise (SD = 12.5). International experts had a mean age of 26.7 years (SD = 14.2), were 70% female (*N* = 7), primarily from Canada and the United Kingdom, with backgrounds in research and education/training, particularly in public and population health. They were typically affiliated with not-for-profit organisations and averaged 18.4 years of expertise (SD = 10.5). The sample had a high proportion of people identifying as female, which is consistent with the distribution of the health, social care, and community-sector workforces in Australia, although the underlying reasons for this distribution in our sample cannot be verified.

**Table 1:** Participant characteristics of the Delphi experts.

Characteristic	Australian experts	International experts
Sample		
Round one	36	11
Round two	33	10
Age		
Mean (SD)	30.31 (11.79)	26.73 (14.15)
Gender		
Female	25	7
Male	11	3
Non-binary	–	1
Australian location		
Australian Capital Territory	1	
New South Wales	9	
Queensland	16	
South Australia	1	
Victoria	8	
Western Australia	1	
International location		
Canada		5
Europe		1
United Kingdom		4
United States		1
Expertise*		
Aged and Community Care Provider	2	
Community Based Organisation	1	
Consumer or Carer of Person with Lived Experience	2	4
Educator and Training	6	5
Health Service Provider	7	1
Peak Body Representative	3	
Policy/Governance/Governmental/Commissioning	5	2
Researcher	7	5
Social Prescribing/Link Worker	5	
Other		1
Expertise years		
Mean (SD)	17.19 (12.51)	18.36 (10.46)
Educational background/area*		
Health professions (i.e., Medicine, Nursing, Pharmacy, Psychiatry, Psychology, Allied Health, Health Service Provider)	22	3
Public and Population Health (i.e., Public Health, Epidemiology)	13	10
Social and Community Work (i.e., Social Work, Community Engagement, Public Service Advocate, Social Policy)	21	8
Other Disciplines (i.e., Economics, Management, Education, Sociology)	11	6
Lived Experience	8	5
Other^a^	6	2
Expertise type**		
Healthcare provider/practitioner	13	2
Researchers/academics	7	4
Government/policy/peak body	7	–
Consumers/service users	3	–
Multiple roles (provider-Researcher etc.)	6	5
Affiliation*		
Industry	4	
Community Pharmacy	1	
Non-Government Organisation	10	2
University	4	2
Primary Health Network	1	1
Peak Body	3	
Private	1	
Not for Profit	8	3
Other^b^	-	1

*Multiple selections possible. **Participants were categorised based on their primary professional role to assess diversity of expertise. Multiple categories possible for international participants serving in dual roles.

a Responses for Australians included “RTO”, “sport science, adventure”, “Health systems research; Health Policy” and responses for Internationals included “Public health and social prescribing consultant and facilitator”.

b Responses included “health geography, policy, evaluation”.

### 3.2 Round one

**Figure 1:**
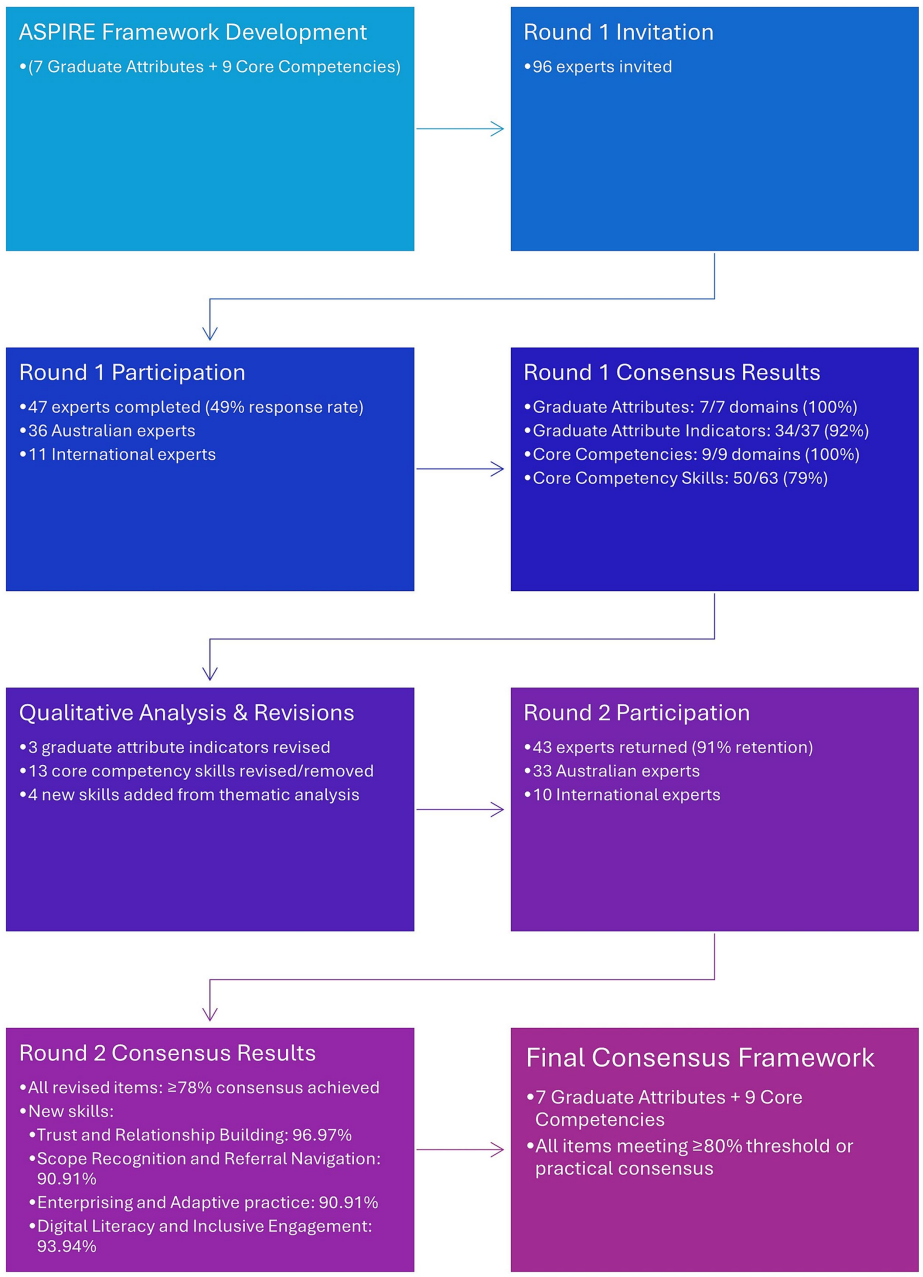
Delphi study flow and consensus results.

Figure 1 illustrates the flow of the study. Round 1 achieved high-level consensus on all seven graduate attribute domains and all nine core competency domains. Among the detailed indicators and skills, 34 of 37 graduate attribute indicators (92%) and 50 of 63 competency-associated skills (79%) reached the 80% consensus threshold. The three graduate attribute indicators and 13 associated skills that did not reach consensus primarily reflected concerns about role scope boundaries, the emphasis on evidence versus lived experience, and resource navigation responsibilities (detailed in Table 2).

**Table 2:** Items that did not meet consensus in Round 1.

Item	N Australian Experts who selected “Somewhat” or “Completely Agree”	N International Experts who selected “Somewhat” or “Completely Agree”	Concerns	Decision
Graduate attributes				
4.2 Evidence-based practice and evaluation	27 (79.41%)	8 (72.72%)	Evidence should prioritise individual and community experiences over academic research Evaluation responsibilities may exceed link worker role scope Evaluation expertise better suited to dedicated analysts and specialists	Changed to “Evidence-Informed Practice” and text was revised to better reflect feedback
4.4 Future-focused strategic planning	27 (79.41%)	8 (72.72%)	Strategic planning better aligned with organisational/management roles Scope exceeds day-to-day link worker responsibilities Time constraints limit capacity for strategic work	Changed to “Supporting Community-Focused Initiatives” and text was revised to better reflect feedback
5.5 Resilience and persistence	27 (79.41%)	9 (81.82%)	System change advocacy beyond standard link worker scope Pay grade misalignment with level of responsibility Need to distinguish between direct service and system change roles	Changed to “Resilience and Adaptability” and text was revised to better reflect feedback
Core competencies				
1.6 Funding and resource navigation	25 (78.13%)	9 (81.82%)	Scope exceeded link worker role Implementation clarity needed Resource limitations not acknowledged	Maintained skill label but text was revised to better reflect scope and to clarify role
7.4 Impact Reporting 7.8 Systemic Change Impact Evaluation	21 (67.74%) 22 (70.97%)	8 (72.72%) 7 (63.63%)	Clarity needed about link worker role in system change Data collection expectations too broad Implementation support requirements unclear	Items combined and revised to “Evidence Collection for System Impact”
1.5 Policy alignment and strategic integration 8.4 Policy engagement and influence	24 (75.01%) 24 (77.42%)	8 (72.72%) 8 (72.72%)	Strategic role vs. operational role clarity needed Scope of policy engagement unclear Level of responsibility concerns	Revised Version (to go in *Supportive Partnerships and System Change* Competency): “Awareness and Communication of Policy Impacts”
4.6 Interest balancing and conflict resolution	25 (78.13%)	9 (81.82%)	Role boundaries in conflict resolution unclear Scope of intervention too broad Need for clear referral pathways	Revised to “Navigating Complex Situations”
9.4 Climate-resilient health planning 9.5 Environmental health education 9.6 Sustainable community	17 (54.84%) 22 (70.97%) 22 (70.97%)	8 (72.72%) 9 (81.82%) 8 (72.72%)	Strategic environmental planning beyond scope Need for focus on practical local level Role boundaries unclear	Revised to “Environmental Health Awareness”
7.2 Evaluation design	24 (77.42%)	8 (72.72%)	Beyond scope of a link worker	Moved to a separate advanced link worker competency
7.3 Outcome measurement and analysis	24 (77.42%)	8 (72.72%)	Beyond scope of a link worker	Moved to a separate advanced link worker competency
7.6 Data-driven advocacy	23 (74.2%)	9 (81.82%)	Beyond scope of a link worker	Moved to a separate advanced link worker competency
8.6 Systems-level impact evaluation	20 (64.51%)	8 (72.72%)	Beyond scope of a link worker	Moved to a separate advanced link worker competency
3.6 Addressing the Digital Divide	28 (87.5%)	8 (72.72%)	International experts expressed concerns that digital literacy support should be a specialised referral service rather than part of the link worker role Australian experts emphasised digital literacy as crucial and saw supporting digital skills development as a necessary part of the role	No action required as Australian consensus reached
7.5 Feedback Integration	25 (80.65%)	8 (72.72%)	International experts suggested feedback integration should be handled by dedicated researchers and evaluation specialists Australian experts identified feedback collection and integration as essential for informing service delivery and development	No action required as Australian consensus reached
8.5 Local needs assessment and reporting	29 (93.54%)	8 (72.72%)	International experts believed needs assessment and reporting should be managed at leadership level rather than by link workers Australian experts highlighted the importance of link workers conducting standardised needs assessments to build evidence across regions	No action required as Australian consensus reached

Perspectives were analysed collectively for the primary consensus assessment, consistent with standard Delphi methodology. Australian and international responses were examined descriptively as a secondary step to validate alignment between national and global expertise and to ensure the resulting standards were coherent with international models of social prescribing. At the completion of round one, consensus (≥80% selecting “Completely Agree” or “Somewhat Agree”) was reached on all 7 graduate attributes (Ethical Practitioner, Inclusive and Respectful, Effective Communicator, Critical Thinker and Problem Solver, Systemic Change Catalyst, Community Empowerment and Collaboration, Reflective and Lifelong Learner) and all 9 core competencies domains (Working across Australian Health and Social Systems, Working with people on what matters, Resource Navigation and Management, Community Development and Social Capital Building, Culturally Safe and Inclusive Practice, Safe and Effective Practice, Data Management and Evaluation, Supportive Partnerships and System Change, Integration of Environment and Contextual Factors) (see Supplementary material for detailed results). Among the 37 key indicators of graduate attributes, 34 reached consensus, and 50 of the 63 associated skills of the core competencies reached consensus. International experts (recruited for benchmarking) showed similar patterns, with 35 key indicators of graduate attributes reaching consensus. The two remaining attributes also did not reach consensus among the Australian group. Of the 12 associated skills that did not reach consensus among international experts, 9 also did not reach consensus with the Australian group. Table 2 lists all the items that did not meet consensus and the outcomes for each one.

74% participants left at least one comment. In total, there were 293 comments across 19 feedback options. Analysis of Round 1 qualitative feedback revealed several significant gaps in the original competency framework that led to the development of four new skills. First, participants emphasised that relationship building was foundational to effective social prescribing but was not explicitly captured, with feedback noting “a relational way of working and building trust is imperative… the relational aspects and trust/empathy elements are missing.” This led to the development of the “Trust and Relationship Building” skill. Second, concerns about role definition emerged strongly, with participants questioning “what distinguishes a Link worker from a Social worker” with the suggestion that “link workers should focus on connecting people with community resources to support their well-being, rather than acting as therapists or clinical practitioners.” This informed the development of the “Scope Recognition and Referral Navigation” skill. Third, participants frequently discussed the need for creativity within resource constraints, noting how service delivery “really depends upon the funding and case load of the link worker,” leading to the “Enterprising and Adaptive Practice” skill. Finally, analysis revealed significant concerns about digital access and inclusion, with participants noting that while “digital literacy is pretty crucial,” there was also a need to consider those who cannot engage with technology, leading to the development of the “Digital Literacy and Inclusive Engagement” skill. These new skills were developed to address these identified gaps while maintaining alignment with the broader objectives of social prescribing, strengthening the framework by explicitly addressing core elements of practice that were previously implicit or absent.

### 3.3 Round two

The results from Round 2 are presented in Table 3. 63.0% participants left at least one comment. In total, there were 554 comments across 13 feedback options. In Round 2 all the items reached consensus. Two items—‘Funding and Resource Navigation’ (78.79%) and ‘Environmental Health Awareness’ (78.79%)—achieved consensus slightly below the predetermined 80% threshold, they were within the 2% margin that previous social prescribing Delphi studies have considered acceptable (6). Crucially, qualitative feedback analysis revealed a distinct pattern from Round 1 items that failed to reach consensus. Unlike Round 1, where qualitative feedback indicated fundamental concerns about scope and role boundaries requiring substantial revision, Round 2 feedback focused primarily on implementation considerations rather than conceptual disagreement. For example, feedback on ‘Funding and Resource Navigation’ centred on clarifying practical application rather than questioning its inclusion in the framework. Similarly, comments on ‘Environmental Health Awareness’ focused on level of emphasis and practical implementation rather than fundamental changes to the concept. This pattern of feedback, combined with the high level of quantitative agreement, supported the retention of these items without need for further rounds. For educators and policymakers, these items with marginally lower consensus (78.79%) should be understood as competencies requiring particular attention to organisational context and resource availability during implementation, with expected variation in how they are enacted across different practice settings.

**Table 3:** Results from Round 2.

Original	Revised	*N*	Consensus N (%)	Completely Agree	Somewhat Agree	Neutral	Somewhat Disagree	Completely Disagree
Australian experts								
Graduate attributes								
4.2 Evidence-based practice and evaluation	4.2 Evidence-Informed Practice	33	31 (93.94%)	19 (57.58%)	12 (36.36%)	1 (3.03%)	0 (0.00%)	1 (3.03%)
4.4 Future-focused strategic planning	4.4 Supporting Community-Focused Initiatives	33	31 (93.94%)	16 (48.48%)	15 (45.45%)	1 (3.03%)	0 (0.00%)	1 (3.03%)
5.5 Resilience and persistence	5.5 Resilience and Adaptability	33	30 (90.91%)	18 (54.55%)	12 (36.36%)	1 (3.03%)	1 (3.03%)	1 (3.03%)
Core competencies								
1.6 Funding and resource navigation	1.6 Funding and resource navigation	33	26 (78.79%)	11 (33.33%)	15 (45.45%)	2 (6.06%)	3 (9.09%)	2 (6.06%)
7.4 Impact Reporting 7.8 Systemic Change Impact Evaluation	7.2 Evidence Collection for System Impact	33	30 (90.91%)	21 (63.64%)	9 (27.27%)	0 (0.00%)	2 (6.06%)	1 (3.03%)
1.5 Policy Alignment and Strategic Integration 8.4 Policy Engagement and Influence	8.4 Awareness and Communication of Policy Impacts	33	29 (87.88%)	18 (54.55%)	11 (33.33%)	1 (3.03%)	1 (3.03%)	2 (6.06%)
4.6 Interest Balancing and Conflict Resolution	4.6 Navigating Complex Situations	33	28 (84.85%)	18 (54.55%)	10 (30.30%)	4 (12.12%)	0 (0.00%)	1 (3.03%)
9.4. Climate-Resilient Health Planning 9.5. Environmental Health Education 9.6. Sustainable Community Initiative Support	9.4 Environmental Health Awareness	33	26 (78.79%)	18 (54.55%)	8 (24.24%)	3 (9.09%)	3 (9.09%)	1 (3.03%)
New skills								
	2.8 Trust and Relationship Building	33	32 (96.97%)	26 (78.79%)	6 (18.18%)	0 (0.00%)	0 (0.00%)	1 (3.03%)
	6.8 Scope Recognition and Referral Navigation	33	30 (90.91%)	20 (60.61%)	10 (30.30%)	1 (3.03%)	1 (3.03%)	1 (3.03%)
	3.9 Enterprising and Adaptive Practice	33	30 (90.91%)	19 (57.58%)	11 (33.33%)	0 (0.00%)	2 (6.06%)	1 (3.03%)
	5.4 Digital Literacy and Inclusive Engagement	33	31 (93.94%)	19 (57.58%)	12 (36.36%)	1 (3.03%)	0 (0.00%)	1 (3.03%)
International experts								
Graduate attributes								
4.2 Evidence-based practice and evaluation	4.2 Evidence-Informed Practice	10	9 (90.00%)	5 (50.00%)	4 (40.00%)	0 (0.00%)	1 (10.00%)	0 (0.00%)
4.4 Future-focused strategic planning	4.4 Supporting Community-Focused Initiatives	10	9 (90.00%)	4 (40.00%)	5 (50.00%)	1 (10.00%)	0 (0.00%)	0 (0.00%)
5.5 Resilience and persistence	5.5 Resilience and Adaptability	10	10 (100.00%)	5 (50.00%)	5 (50.00%)	0 (0.00%)	0 (0.00%)	0 (0.00%)
Core competencies								
1.6 Funding and resource navigation	1.6 Funding and resource navigation	10	7 (70.00%)	5 (50.00%)	2 (20.00%)	0 (0.00%)	3 (30.00%)	0 (0.00%)
7.4 Impact Reporting 7.8 Systemic Change Impact Evaluation	7.2 Evidence Collection for System Impact	10	9 (90.00%)	8 (80.00%)	1 (10.00%)	1 (10.00%)	0 (0.00%)	0 (0.00%)
1.5 Policy Alignment and Strategic Integration 8.4 Policy Engagement and Influence	8.4 Awareness and Communication of Policy Impacts	10	9 (90.00%)	7 (70.00%)	2 (20.00%)	1 (10.00%)	0 (0.00%)	0 (0.00%)
4.6 Interest Balancing and Conflict Resolution	4.6 Navigating Complex Situations	10	10 (100.00%)	7 (70.00%)	3 (30.00%)	0 (0.00%)	0 (0.00%)	0 (0.00%)
9.4. Climate-Resilient Health Planning 9.5. Environmental Health Education 9.6. Sustainable Community Initiative Support	9.4 Environmental Health Awareness	10	9 (90.00%)	5 (50.00%)	4 (40.00%)	0 (0.00%)	1 (10.00%)	0 (0.00%)
New skills								
	2.8 Trust and Relationship Building	10	10 (100.00%)	7 (70.00%)	3 (30.00%)	0 (0.00%)	0 (0.00%)	0 (0.00%)
	6.8 Scope Recognition and Referral Navigation	9	9 (100.00%)	8 (88.89%)	1 (11.11%)	0 (0.00%)	0 (0.00%)	0 (0.00%)
	3.9 Enterprising and Adaptive Practice	9	7 (77.78%)	5 (55.56%)	2 (22.22%)	2 (22.22%)	0 (0.00%)	0 (0.00%)
	5.4 Digital Literacy and Inclusive Engagement	9	7 (77.78%)	6 (66.67%)	1 (11.11%)	2 (22.22%)	0 (0.00%)	0 (0.00%)

Round 2 results showed strong alignment between Australian and international experts across all revised items. The revised graduate attributes - Evidence-Informed Practice, Supporting Community-Focused Initiatives, and Resilience and Adaptability - achieved consensus above 90% from both groups. Among core competencies, Evidence Collection for System Impact, Awareness and Communication of Policy Impacts, and Navigating Complex Situations similarly achieved strong consensus. Environmental Health Awareness received slightly higher support from international experts (90.00%) than Australian experts (78.79%), reflecting different contextual emphases. The newly proposed skills were well-received by both groups (see Figure 1).

Analysis of Round 2 qualitative feedback focused on refinement and implementation considerations. Participants emphasised the importance of organisational support and clear role boundaries, particularly for Funding and Resource Navigation, noting that “some aspects should be organization-led rather than link worker responsibility.” The newly added skills received strong endorsement, with particular emphasis on Trust and Relationship Building as “foundational to effective social prescribing” while requiring “clear boundaries and scope documents” for implementation. For Environmental Health Awareness, feedback highlighted the need to “separate into different levels (awareness vs. implementation)” while maintaining the core competency. The newly added skills received strong endorsement with particular emphasis on their practical application. For example, feedback on Trust and Relationship Building noted that while “relationship building was foundational to effective social prescribing,” there needed to be “clear boundaries and scope documents” to support implementation. Similarly, commentary on Digital Literacy and Inclusive Engagement acknowledged its importance while emphasising the need for “IT support” and consideration of contexts where “technological barriers” could not always be addressed. This feedback pattern reinforced the shift from Round 1’s fundamental concerns to Round 2’s focus on practical implementation considerations.

### 3.4 Refined educational standards

**Figure 2:**
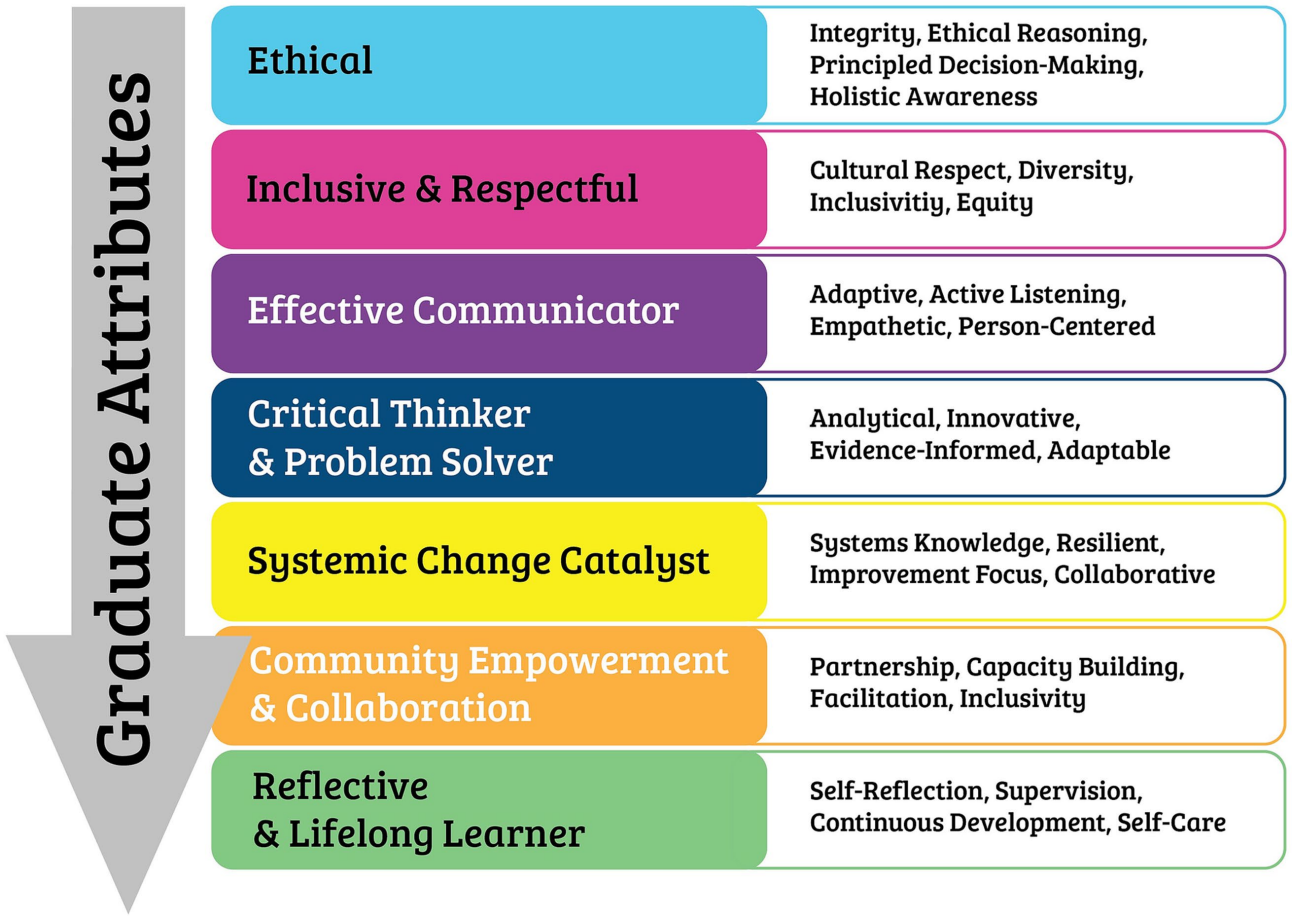
The graduate attributes of a social prescribing link worker.

**Figure 3:**
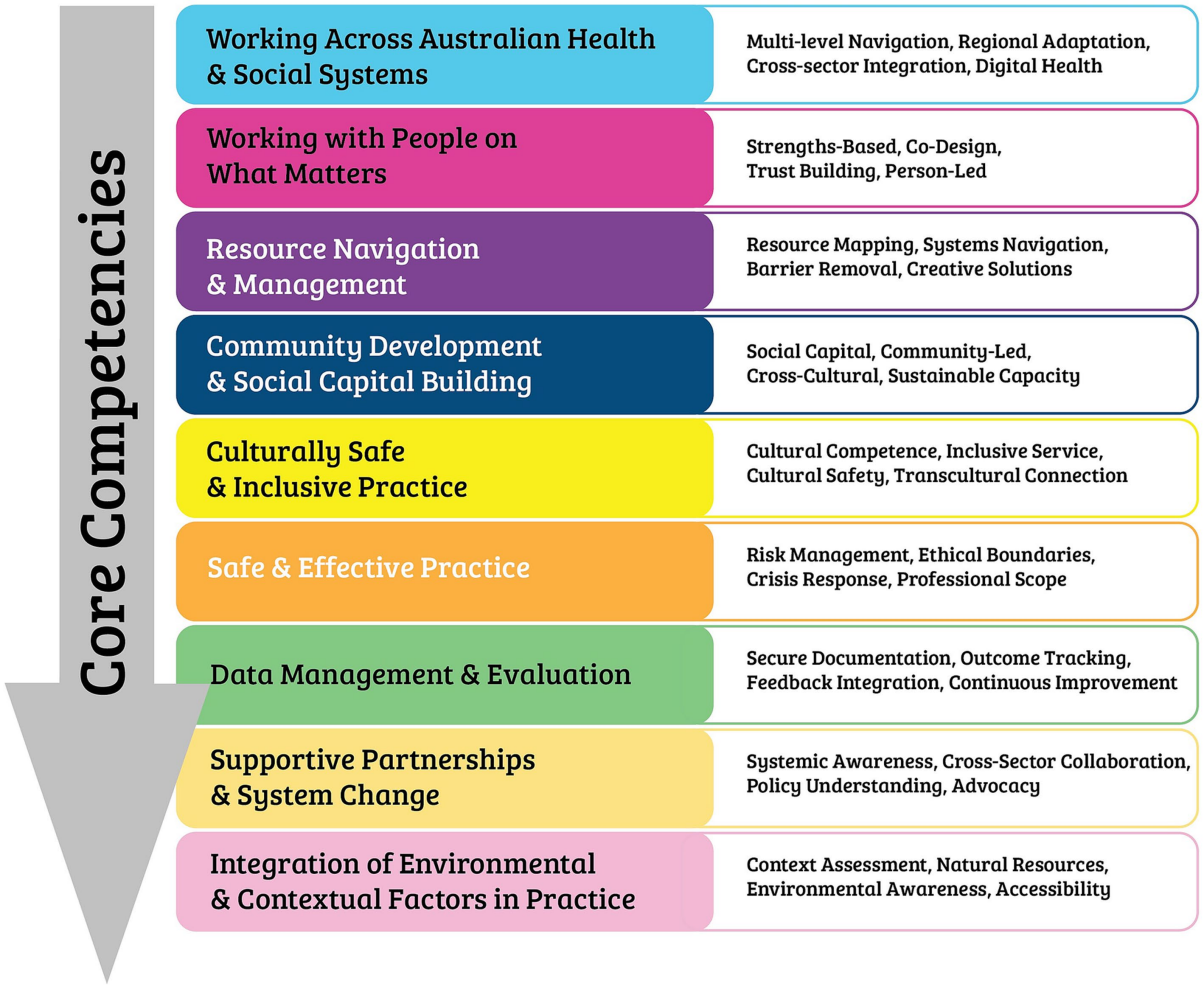
The core competencies of a social prescribing link worker.

Following the two-round Delphi process, consensus was reached on the educational standards for Australian social prescribing Link Workers. The final framework comprises seven graduate attributes that define the professional characteristics expected of qualified link workers, and nine core competencies that detail the specific skills and knowledge required for effective practice (Figures 2, 3) These standards provide a foundation for curriculum development, professional training programmes, and quality assurance in social prescribing link worker education across Australia. The complete final standards with detailed indicators and skills are provided in Supplementary material.

## 4 Discussion

This Delphi study has developed the first consensus-based educational standards framework for social prescribing Link Workers in Australia, achieving strong consensus on seven graduate attributes and nine core competencies, among diverse stakeholders. These standards represent an important foundational step toward professionalising the social prescribing workforce, addressing existing fragmentation and variability in link worker training and practice through proposing a common language and national benchmarks. The framework provides a consensus-based foundation for developing training programs, qualifications, and professional development pathways. However, these standards represent expert agreement on educational priorities rather than empirically validated training outcomes, and require testing and refinement through implementation in educational settings and evaluation of graduate preparedness before they can be considered established standards. As with all consensus-based standards, these reflect the perspectives of those currently shaping the field and should be understood as provisional, evolving alongside empirical evidence and practice.

High levels of consensus were achieved across most domains, with over 90% agreement from both Australian and international experts, indicating broad stakeholder support for these standards. The study makes significant contributions to both national workforce development and international knowledge. While countries like the United Kingdom have advanced large-scale link worker implementation, few have articulated comprehensive education standards through systematic consensus processes. This framework demonstrates how standards can be co-designed to reflect national priorities while remaining aligned with international evidence, offering a replicable model for other health systems seeking to integrate social prescribing within diverse cultural, geographic, and workforce contexts.

The Australian educational standards demonstrate both convergence with and differentiation from international frameworks, reflecting contextual adaptation to Australia’s unique healthcare environment (see Table 4). While sharing foundational elements such as person-centred care and community connection with established frameworks like England’s NHS competencies and Canada’s CISP model (9, 10), the Australian standards introduce several distinctive features. Most notably, the framework places greater emphasis on multi-funder healthcare navigation as a core competency, reflecting Australia’s complex blend of Medicare, NDIS, My Aged Care, and state-based services -a challenge less prominent in single-payer systems like England’s NHS. The Australian framework places greater emphasis on systemic change advocacy and policy engagement compared to more operationally focused international approaches. Unlike Canada’s approach of establishing cultural safety as a distinct competency domain, Australia integrates First Nations perspectives throughout multiple competency areas rather than treating cultural safety as a separate component. While core social prescribing principles of community connection and person-centred care remain consistent internationally, the Australian standards reflect contextual adaptation to the nation’s unique healthcare governance structures, cultural landscape, and implementation challenges.

**Table 4:** Comparison of international social prescribing link worker frameworks.

Framework element	England (NHS)	Canada (CISP)	Australia (ASPIRE)
Framework structure	4 core competencies: • Engage & connect with people • Enable & support people • Community development • Safe & effective practice	Link Worker Competency Framework + Training Roadmap developed	7 graduate attributes and 9 core competencies
Training delivery	12 mandatory e-learning modules via Personalised Care Institute	Training Roadmap with resources for different stakeholder groups	Educational standards for flexible delivery pathways. Additional training delivery to be developed.
National coordination	NHS England centrally mandated	Canadian Institute for Social Prescribing (CISP) - national network	Australian Social Prescribing Institute (ASPIRE)
Development method	Established through NHS policy implementation	Stakeholder consultation and survey-based development	Delphi consensus study with international benchmarking
Cultural integration	Separate cultural competence components	Cultural Safety and Competence as distinct domain with Indigenous-specific training resources	First Nations perspectives integrated across multiple competency domains (cultural safety, environmental health, community empowerment)

Beyond contributing to Australia’s social prescribing development, both the finalised standards and the Delphi approach used to generate them offer a useful model for other health systems seeking to design their own context-specific link worker competencies. The integration of global evidence, international benchmarking, and national stakeholder consensus through the Delphi process demonstrates how countries can adapt international best practices while maintaining local relevance. This approach may be particularly valuable for health systems with similar challenges around cultural diversity, geographic complexity, or multi-funder environments, providing a replicable framework for systematic workforce development in social prescribing.

### 4.1 Policy implications

These consensus-based educational standards provide a foundation for policy development that addresses social prescribing workforce gaps in Australia’s evolving healthcare landscape. The framework proposes link workers as a distinct professional category within Australia’s existing health workforce structure, complementing rather than duplicating established roles such as social work, nursing, and allied health. The standards align directly with national policy priorities outlined in the National Preventive Health Strategy 2021–2030 (33) and the Primary Health Care 10-Year Plan 2022–2032 (34), both of which advocate for community-based interventions to address social determinants of health.

Similarly to other high-income countries, Australia faces mounting healthcare challenges that demand innovative workforce solutions. Australia’s rising burden of chronic conditions among young people (2), increasing mental health challenges in young adults (3), and an ageing population (4) are placing unprecedented pressure on clinical services (2–4). Simultaneously, workforce shortages across general practice, nursing, and mental health represent critical undersupply areas underscoring the urgency of deploying a non-clinical workforce to support early intervention and community-based care (5). By providing consensus on the educational preparation required for link workers, the framework offers a foundation that could support efforts to redirect non-medical needs away from overstretched clinical services towards appropriate community-based supports. This systematic approach to workforce development provides commissioners with a credible tool to guide investment decisions and quality assurance processes, enabling evidence-based resource allocation and performance monitoring. These standards enable rapid deployment of a non-clinical workforce capable of addressing psychosocial factors while reducing pressure on clinical services.

The framework addresses Australia’s unique implementation challenges through competencies specifically designed for the nation’s multi-funder healthcare environment, geographic diversity, and cultural complexity. Unlike single-payer systems, Australia’s blend of federal and state governance, Medicare, NDIS, My Aged Care, and community funding streams requires link workers skilled in complex system navigation. The standards’ emphasis on cultural safety for First Nations peoples and rural-remote adaptability reflects Australia’s distinctive demographic and geographic characteristics.

Implementation science perspectives highlight how these standards address fundamental barriers to social prescribing adoption. Qualitative feedback revealed concerns about role duplication with social work; however, comparison with the Australian Social Work Education and Accreditation Standards demonstrates distinct yet complementary scopes of practice (53). Rather than duplicating existing professional roles, link workers address a distinct gap in the healthcare workforce. While social workers typically manage complex cases requiring statutory intervention, therapeutic support, or intensive case management, link workers operate with a broader population-level approach, connecting people to community resources and non-clinical supports. For example, while a social worker might provide ongoing case management for someone experiencing domestic violence, a link worker would connect someone to community gardening groups, walking clubs, or volunteering opportunities to address social isolation. This differentiation enables social workers to focus on their specialised scope while link workers address the broader population’s social determinants of health through community engagement. In this way, link workers complement rather than duplicate social work roles, addressing a gap at a lower scope of practice but with a strong orientation toward community building and wellbeing. This differentiation also points toward the value of conceptualising link worker practice within a broader framework of intervention levels and intensity—a potential direction for future work on social prescribing typologies.

The standards provide policymakers, funders, and commissioning bodies with a consensus-based framework for workforce planning and quality assurance. By proposing clear competency expectations and training pathways, the framework offers a foundation for systematic scaling of social prescribing across diverse healthcare settings, with the aim of promoting consistent quality and outcomes pending validation through implementation. If validated through implementation research, such standards could support efforts to leverage social prescribing as a cost-effective and environmentally conscious strategy for addressing healthcare capacity constraints and improving population health outcomes through community-based rather than clinic-based interventions.

### 4.2 Strengths and limitations

The strengths of this study include robust mixed-methods Delphi methodology combining quantitative consensus measurement with qualitative feedback analysis. The diverse stakeholder representation across healthcare disciplines, research backgrounds, and practice settings strengthened the validity of consensus. High retention rates between rounds (91%) and substantial qualitative engagement (74% of Round 1 participants providing comments) indicate strong participant investment in the standards development process. The integration of international benchmarking provided valuable external validation while maintaining focus on Australian contextual requirements.

Limitations include potential sampling bias, with 78% female participation among Australian experts, which may not fully represent the diversity of perspectives in the emerging field. While we did not analyse consensus patterns by participant gender, it is possible that the gender distribution may have influenced consensus levels on competencies emphasising relational and care-oriented aspects of practice. Ethnicity and cultural background were not captured because expertise was the primary recruitment criterion. As social prescribing grows nationally and globally, future validation should ensure that a more diverse demographic representation is included to strengthen applicability across varied populations and workforce contexts. As an emerging field, the definition of “expertise” was necessarily broad, which may have included varied levels of social prescribing-specific knowledge. Additionally, the sample comprised predominantly social prescribing advocates and practitioners, which may have introduced positive bias towards comprehensive standards. Participants’ professional investment in advancing social prescribing could have influenced consensus levels, particularly for competencies expanding the link worker role. However, this limitation is inherent to developing standards for emerging fields where expertise necessarily derives from advocacy and practice experience. The inclusion of critical feedback and items that failed to reach consensus demonstrates that participants engaged critically rather than endorsing all proposed standards uncritically.

The sampling approach, while achieving diversity across expertise types and geographical regions, cannot claim statistical representativeness of all potential stakeholders in Australian social prescribing. The reliance on professional networks and purposive sampling may have systematically excluded perspectives from practitioners working in very remote areas, those from culturally and linguistically diverse backgrounds who may face language barriers, and community organisations without established connections to social prescribing networks. These limitations may have influenced consensus results by over-representing perspectives from well-connected, professionally established individuals and organisations with existing social prescribing experience. However, the high consensus levels achieved (>90% for most items) and the integration of international benchmarking suggest that the standards reflect broadly applicable principles rather than narrow, context-specific perspectives. Future validation research should deliberately seek input from underrepresented stakeholder groups, particularly from remote and very remote communities, culturally and linguistically diverse populations, and emerging practitioners new to the field.

The consensus thresholds, while established in literature, remain somewhat arbitrary, and the standards require validation through implementation and evaluation before being considered definitively established. A limitation is that the study focused on professional and expert perspectives, and future research should incorporate direct input from community members and service users who are the intended recipients of social prescribing services Future research should examine how these standards perform in diverse educational and practice settings, measure their impact on workforce preparation and practice quality, and incorporate perspectives from community members and service users who are direct recipients of social prescribing services.

## 5 Conclusion

This Delphi study has developed the first consensus-based educational standards framework for social prescribing link workers in Australia. Through a rigorous two-round process involving diverse stakeholders, strong consensus was achieved on seven graduate attributes and nine core competencies that define the knowledge, skills, and professional characteristics required for effective link worker practice. The high levels of agreement (over 90% for most items) indicate broad stakeholder support for the framework’s approach to professionalising this emerging workforce. The framework effectively balances comprehensive professional capabilities with practical implementation considerations, distinguishing link workers from other healthcare roles while emphasising their unique function in connecting clinical and community contexts. By creating clarity around the scope and role boundaries of link worker practice, these standards address a fundamental implementation barrier while providing clear pathways for professional recognition and career development.

These consensus-based educational standards provide a foundation for developing the infrastructure necessary to transition social prescribing from fragmented pilot programs toward systematic implementation. Once validated through implementation and refinement, a framework emphasising evidence-informed practice, cultural safety, and community empowerment could position Australia to scale social prescribing as an accountable and sustainable strategy for addressing social determinants of health. However, the standards represent a starting point requiring validation through implementation, evaluation, and ongoing refinement as the field continues to evolve. The next phase of this research will involve qualitative interviews and focus groups with community members, service users, and diverse stakeholder groups who were not included in this Delphi process, to evaluate and refine the framework based on end-user perspectives. Additionally, future work should focus on pilot testing these standards in educational settings, developing appropriate assessment methods, and evaluating their impact on workforce quality and service outcomes. The framework’s emphasis on evidence-based practice and continuous evaluation ensures that as social prescribing expands it adapts to emerging healthcare challenges and opportunities.

## Data Availability

The raw data supporting the conclusions of this article will be made available by the authors, without undue reservation.
