# Supplementary material for: Educational standards for Australian social prescribing link workers: a modified Delphi study
Source: Front Public Health. 2026 Feb 19;14:1754668. doi: 10.3389/fpubh.2026.1754668 (PMC12960570; doi:10.3389/fpubh.2026.1754668)
Supplement: Supplementary file 1 [file Data_Sheet_1.docx]

# Round 1 Results for Australian Experts

|  | **N** | **Round 1 Results***† | **N Completely Agree (5)** | **N Somewhat Agree (4)** | **N Neither Agree nor Disagree (3)** | **N Somewhat Disagree (2)** | **N Completely Disagree (1)** |
| --- | --- | --- | --- | --- | --- | --- | --- |
| **Graduate Attributes** |  |  |  |  |  |  |  |
| 1. Ethical Practitioner | 36 | 36 (100%) | 30 (83.33%) | 6 (16.67%) | 0 (0%) | 0 (0%) | 0 (0%) |
| 1.1 Professional Integrity | 36 | 36 (100%) | 33 (91.67%) | 3 (8.33%) | 0 (0%) | 0 (0%) | 0 (0%) |
| 1.2 Ethical Awareness and Reasoning | 36 | 35 (97.23%) | 20 (55.56%) | 15 (41.67%) | 0 (0%) | 1 (2.78%) | 0 (0%) |
| 1.3 Ethical Decision-Making | 36 | 34 (94.45%) | 23 (63.89%) | 11 (30.56%) | 1 (2.78%) | 1 (2.78%) | 0 (0%) |
| 1.4 Legal and Ethical Compliance | 36 | 34 (94.45%) | 24 (66.67%) | 10 (27.78%) | 2 (5.56%) | 0 (0%) | 0 (0%) |
| 1.5 Holistic Awareness | 36 | 31 (86.11%) | 23 (63.89%) | 8 (22.22%) | 4 (11.11%) | 1 (2.78%) | 0 (0%) |
| 2. Inclusive and Respectful | 36 | 34 (94.45%) | 33 (91.67%) | 1 (2.78%) | 0 (0%) | 1 (2.78%) | 1 (2.78%) |
| 2.1 Cultural Respect | 36 | 36 (100%) | 28 (77.78%) | 8 (22.22%) | 0 (0%) | 0 (0%) | 0 (0%) |
| 2.2 Cultural Awareness | 36 | 33 (91.66%) | 26 (72.22%) | 7 (19.44%) | 2 (5.56%) | 1 (2.78%) | 0 (0%) |
| 2.3 Cultural Humility | 36 | 33 (91.66%) | 28 (77.78%) | 5 (13.89%) | 2 (5.56%) | 1 (2.78%) | 0 (0%) |
| 2.4 Inclusive Mindset | 36 | 35 (97.23%) | 31 (86.11%) | 4 (11.11%) | 1 (2.78%) | 0 (0%) | 0 (0%) |
| 2.5 Commitment to Equity | 36 | 34 (94.45%) | 33 (91.67%) | 1 (2.78%) | 2 (5.56%) | 0 (0%) | 0 (0%) |
| 3. Effective Communicator | 34 | 33 (97.06%) | 31 (91.18%) | 2 (5.88%) | 0 (0%) | 0 (0%) | 1 (2.94%) |
| 3.1 Adaptive Communication | 34 | 33 (97.06%) | 29 (85.29%) | 4 (11.76%) | 1 (2.94%) | 0 (0%) | 0 (0%) |
| 3.2 Active Listening and Deep Engagement | 34 | 34 (100%) | 31 (91.18%) | 3 (8.82%) | 0 (0%) | 0 (0%) | 0 (0%) |
| 3.3 Empathetic and Person-Centred Communication | 34 | 34 (100%) | 29 (85.29%) | 5 (14.71%) | 0 (0%) | 0 (0%) | 0 (0%) |
| 3.4 Management of Sensitive Conversations | 34 | 34 (100%) | 30 (88.24%) | 4 (11.76%) | 0 (0%) | 0 (0%) | 0 (0%) |
| 3.5 Using techniques to support action | 34 | 32 (94.12%) | 25 (73.53%) | 7 (20.59%) | 2 (5.88%) | 0 (0%) | 0 (0%) |
| 4. Critical Thinker and Problem Solver | 34 | 30 (88.24%) | 16 (47.06%) | 14 (41.18%) | 2 (5.88%) | 1 (2.94%) | 1 (2.94%) |
| 4.1 Analytical and Innovative Thinking | 34 | 30 (88.24%) | 14 (41.18%) | 16 (47.06%) | 4 (11.67%) | 0 (0%) | 0 (0%) |
| 4.2 Evidence-based practice and evaluation | 34 | **27 (79.41%)** | 17 (50.00%) | 10 (29.41%) | 5 (14.71%) | 2 (5.88%) | 0 (0%) |
| 4.3 Adaptive problem solving | 34 | 28 (82.35%) | 21 (61.76%) | 7 (20.59%) | 6 (17.65%) | 0 (0%) | 0 (0%) |
| 4.4 Future-focused strategic planning | 34 | **27 (79.42%)** | 13 (38.24%) | 14 (41.18%) | 4 (11.76%) | 3 (8.82%) | 0 (0%) |
| 4.5 Resource gap analysis and development | 34 | 31 (91.18%) | 23 (67.65%) | 8 (23.53%) | 2 (5.88%) | 1 (2.94%) | 0 (0%) |
| 4.6 Inclusive Thinking and Creativity | 34 | 32 (94.12%) | 19 (55.88%) | 13 (38.24%) | 2 (5.88%) | 0 (0%) | 0 (0%) |
| 5. Systemic Change Catalyst | 34 | 31 (91.18%) | 18 (52.94%) | 13 (38.24%) | 2 (5.88%) | 1 (2.94%) | 0 (0%) |
| 5.1 Systems Understanding | 34 | 31 (91.17%) | 19 (55.88%) | 12 (35.29%) | 2 (5.88%) | 1 (2.94%) | 0 (0%) |
| 5.2 Improvement Mindset | 34 | 33 (97.06%) | 18 (52.94%) | 15 (44.12%) | 1 (2.94%) | 0 (0%) | 0 (0%) |
| 5.3 Collective Action Contribution | 34 | 32 (94.12%) | 18 (52.94%) | 14 (41.18%) | 1 (2.94%) | 1 (2.94%) | 0 (0%) |
| 5.4 Intentional and strategic | 34 | 30 (88.24%) | 16 (47.06%) | 14 (41.18%) | 4 (11.76%) | 0 (0%) | 0 (0%) |
| 5.5 Resilience and persistence | 34 | **27 (76.47%)** | 16 (47.06%) | 10 (29.41%) | 6 (17.65%) | 2 (5.88%) | 0 (0%) |
| 6. Community Empowerment and Collaboration | 34 | 31 (91.18%) | 23 (67.65%) | 8 (23.53%) | 3 (8.82%) | 0 (0%) | 0 (0%) |
| 6.1 Health Equity Approach | 34 | 31 (91.18%) | 25 (73.53%) | 6 (17.65%) | 2 (5.88%) | 1 (2.94%) | 0 (0%) |
| 6.2 Community Empowerment and capacity building | 34 | 33 (97.06%) | 27 (79.41%) | 6 (17.65%) | 1 (2.94%) | 0 (0%) | 0 (0%) |
| 6.3 Collaborative partnership development | 34 | 33 (97.06%) | 26 (76.47%) | 7 (20.59%) | 1 (2.94%) | 0 (0%) | 0 (0%) |
| 6.4 Interprofessional collaboration | 34 | 32 (94.11%) | 20 (58.82%) | 12 (35.29%) | 1 (2.94%) | 1 (2.94%) | 0 (0%) |
| 6.5 Facilitation of collaborative decision-making | 34 | 31 (91.18%) | 18 (52.94%) | 13 (38.24%) | 1 (2.94%) | 2 (5.88%) | 0 (0%) |
| 6.6 Supporting positive change | 34 | 31 (91.18%) | 18 (52.94%) | 13 (38.24%) | 2 (5.88%) | 1 (2.94%) | 0 (0%) |
| 7. Reflective and lifelong learner | 34 | 32 (94.11%) | 25 (73.53%) | 7 (20.59%) | 0 (0%) | 1 (2.94%) | 1 (2.94%) |
| 7.1 Evidence-based practice | 34 | 31 (91.18%) | 26 (76.47%) | 5 (14.71%) | 2 (5.88%) | 1 (2.94%) | 0 (0%) |
| 7.2 Reflective practice | 34 | 34 (100%) | 28 (82.35%) | 6 (17.65%) | 0 (0%) | 0 (0%) | 0 (0%) |
| 7.3 Continuous professional development | 34 | 31 (91.18%) | 23 (67.65%) | 8 (23.53%) | 3 (8.82%) | 0 (0%) | 0 (0%) |
| 7.4 Professional supervision, peer learning, and mentorship | 34 | 32 (94.11%) | 26 (76.47%) | 6 (17.65%) | 1 (2.94%) | 1 (2.94%) | 0 (0%) |
| 7.5 Self care and collective resilience | 34 | 33 (97.06%) | 25 (73.53%) | 8 (23.53%) | 1 (2.94%) | 0 (0%) | 0 (0%) |
|  |  |  |  |  |  |  |  |
| **Core Competencies** |  |  |  |  |  |  |  |
| 1. Working across Australian Health and Social Systems | 32 | 28 (87.5%) | 22 (68.75%) | 6 (18.75%) | 3 (9.38%) | 1 (3.13%) | 0 (0%) |
| 1.1 Multi-level system understanding | 32 | 27 (84.38%) | 17 (53.13%) | 10 (31.25%) | 2 (6.25%) | 3 (9.38%) | 0 (0%) |
| 1.2 Understand and respond to local and regional needs | 32 | 27 (84.38%) | 15 (46.88%) | 12 (37.50%) | 4 (12.50%) | 1 (3.13%) | 0 (0%) |
| 1.3 Cross-sectoral integration | 32 | 30 (93.75%) | 22 (68.75%) | 8 (25.00%) | 1 (3.13%) | 1 (3.13%) | 0 (0%) |
| 1.4 Rural and remote adaptability | 32 | 30 (93.75%) | 22 (68.75%) | 8 (25.00%) | 2 (6.25%) | 0 (0%) | 0 (0%) |
| 1.5 Policy alignment and strategic integration | 32 | **24 (75.01%)** | 11 (34.38%) | 13 (40.63%) | 5 (15.63%) | 3 (9.38%) | 0 (0%) |
| 1.6 Funding and resource navigation | 32 | **25 (78.13%)** | 13 (40.63%) | 12 (37.50%) | 5 (15.63%) | 2 (6.25%) | 0 (0%) |
| 1.7 Integration with existing programs and schemes | 32 | 27 (84.38%) | 21 (65.63%) | 6 (18.75%) | 5 (15.63%) | 0 (0%) | 0 (0%) |
| 1.8 Digital health integration | 32 | 27 (84.38%) | 20 (62.50%) | 7 (21.88%) | 4 (12.50%) | 1 (3.13%) | 0 (0%) |
| 2. Working with people on what matters | 32 | 32 (100%) | 31 (96.88%) | 1 (3.13%) | 0 (0%) | 0 (0%) | 0 (0%) |
| 2.1 Empathetic and adaptive communication | 32 | 32 (100%) | 30 (93.75%) | 2 (6.25%) | 0 (0%) | 0 (0%) | 0 (0%) |
| 2.2 Inclusive and context-sensitive collaboration | 32 | 31 (96.88%) | 28 (87.50%) | 3 (9.38%) | 1 (3.13%) | 0 (0%) | 0 (0%) |
| 2.3 Comprehensive strengths and needs assessment | 32 | 32 (100%) | 29 (90.63%) | 3 (9.38%) | 0 (0%) | 0 (0%) | 0 (0%) |
| 2.4 Collaborative goal setting and social prescription co-design | 32 | 31 (96.88%) | 29 (90.63%) | 2 (6.25%) | 0 (0%) | 1 (3.13%) | 0 (0%) |
| 2.5 Holistic health enablement | 32 | 31 (96.88%) | 27 (84.38%) | 4 (12.50%) | 0 (0%) | 1 (3.13%) | 0 (0%) |
| 2.6 Adaptable planning | 32 | 32 (100%) | 28 (87.50%) | 4 (12.50%) | 0 (0%) | 0 (0%) | 0 (0%) |
| 2.7 Interprofessional Collaboration | 32 | 30 (93.75%) | 26 (81.25%) | 4 (12.50%) | 2 (6.25%) | 0 (0%) | 0 (0%) |
| 3. Resource Navigation and Management | 32 | 32 (100%) | 26 (81.25%) | 6 (18.75%) | 0 (0%) | 0 (0%) | 0 (0%) |
| 3.1 Community Resource Mapping and Knowledge Management | 32 | 29 (90.63%) | 26 (81.25%) | 3 (9.38%) | 1 (3.13%) | 1 (3.13%) | 1 (3.13%) |
| 3.2 Sustainable Resource Utilisation and Matching | 32 | 30 (93.75%) | 23 (71.88%) | 7 (21.88%) | 2 (6.25%) | 0 (0%) | 0 (0%) |
| 3.3 Resource Optimisation and Development | 32 | 28 (87.5%) | 18 (56.25%) | 10 (31.25%) | 2 (6.25%) | 2 (6.25%) | 0 (0%) |
| 3.4 Systems Navigation | 32 | 30 (93.75%) | 20 (62.50%) | 10 (31.25%) | 0 (0%) | 2 (6.25%) | 0 (0%) |
| 3.5 Barrier Identification and Mitigation | 32 | 31 (96.88%) | 25 (78.13%) | 6 (18.75%) | 1 (3.13%) | 0 (0%) | 0 (0%) |
| 3.6 Addressing the Digital Divide | 32 | 28 (87.5%) | 20 (62.50%) | 8 (25.00%) | 2 (6.25%) | 2 (6.25%) | 0 (0%) |
| 3.7 Collaborative Problem-Solving | 32 | 32 (100%) | 25 (78.13%) | 7 (21.88%) | 0 (0%) | 0 (0%) | 0 (0%) |
| 3.8 Critical Analysis and Innovation | 32 | 27 (84.38%) | 18 (56.25%) | 9 (28.13%) | 4 (12.50%) | 1 (3.13%) | 0 (0%) |
| 4. Community Development and Social Capital Building | 32 | 31 (96.88%) | 22 (68.75%) | 9 (28.13%) | 0 (0%) | 1 (3.13%) | 0 (0%) |
| 4.1 Community Engagement and Empowerment | 32 | 27 (84.38%) | 18 (56.25%) | 9 (28.13%) | 4 (12.50%) | 1 (3.13%) | 0 (0%) |
| 4.2 Social Network and capital building | 32 | 31 (96.88%) | 24 (75.00%) | 7 (21.88%) | 1 (3.13%) | 0 (0%) | 0 (0%) |
| 4.3 Inclusive practice and cross-cultural bridging | 32 | 27 (84.38%) | 22 (68.75%) | 5 (15.63%) | 3 (9.38%) | 2 (6.25%) | 0 (0%) |
| 4.4 Supporting community-led initiatives | 32 | 28 (87.51%) | 19 (59.38%) | 9 (28.13%) | 2 (6.25%) | 2 (6.25%) | 0 (0%) |
| 4.5 Collaborative partnership development | 32 | 29 (90.63%) | 22 (68.75%) | 7 (21.88%) | 2 (6.25%) | 1 (3.13%) | 0 (0%) |
| 4.6 Interest balancing and conflict resolution | 32 | **25 (78.13%)** | 17 (53.13%) | 8 (25.00%) | 6 (18.75%) | 1 (3.13%) | 0 (0%) |
| 4.7 Sustainable community capacity building | 32 | 28 (87.51%) | 16 (50.00%) | 12 (37.50%) | 3 (9.38%) | 0 (0%) | 1 (3.13%) |
| 4.8 Community-led framework implementation | 32 | 29 (90.63%) | 20 (62.50%) | 9 (28.13%) | 1 (3.13%) | 1 (3.13%) | 1 (3.13%) |
| 5. Culturally Safe and Inclusive Practice | 32 | 32 (100%) | 30 (93.75%) | 2 (6.25%) | 0 (0%) | 0 (0%) | 0 (0%) |
| 5.1 Cultural competence development | 32 | 32 (100%) | 26 (81.25%) | 6 (18.75%) | 0 (0%) | 0 (0%) | 0 (0%) |
| 5.2 Cultural safety implementation | 32 | 31 (96.88%) | 27 (84.38%) | 4 (12.50%) | 0 (0%) | 1 (3.13%) | 0 (0%) |
| 5.3 Inclusive Service delivery | 32 | 32 (100%) | 28 (87.50%) | 4 (12.50%) | 0 (0%) | 0 (0%) | 0 (0%) |
| 5.4 Transcultural connectivity and adaptability | 32 | 30 (93.75%) | 26 (81.25) | 4 (12.50%) | 2 (6.25%) | 0 (0%) | 0 (0%) |
| 6. Safe and Effective Practice | 31 | 31 (100%) | 28 (90.32%) | 3 (9.68%) | 0 (0%) | 0 (0%) | 0 (0%) |
| 6.1 Comprehensive risk management | 31 | 30 (96.77%) | 23 (74.19%) | 7 (22.58%) | 1 (3.23%) | 0 (0%) | 0 (0%) |
| 6.2 Navigation of complex situations | 31 | 31 (100%) | 24 (77.42%) | 7 (22.58%) | 0 (0%) | 0 (0%) | 0 (0%) |
| 6.3 Ethical decision-making | 31 | 30 (96.77%) | 26 (83.87%) | 4 (12.90%) | 1 (3.23%) | 0 (0%) | 0 (0%) |
| 6.4 Legal compliance and documentation | 31 | 29 (93.54%) | 23 (74.19%) | 6 (19.35%) | 1 (3.23%) | 1 (3.23%) | 0 (0%) |
| 6.5 Managing crises | 31 | 29 (93.54%) | 22 (70.97%) | 7 (22.58%) | 1 (3.23%) | 0 (0%) | 1 (3.23%) |
| 6.6 Adaptive practice and safety enhancement | 31 | 30 (96.77%) | 23 (74.19%) | 7 (22.58%) | 1 (3.23%) | 0 (0%) | 0 (0%) |
| 6.7 Conflict management | 31 | 29 (93.54%) | 21 (67.74%) | 8 (25.81%) | 1 (3.23%) | 1 (3.23%) | 0 (0%) |
| 7. Data Management and Evaluation | 31 | 29 (93.54%) | 19 (61.29%) | 10 (32.26%) | 1 (3.23%) | 1 (3.23%) | 0 (0%) |
| 7.1 Ethical and Legal Data Management | 31 | 30 (96.77%) | 24 (77.42%) | 6 (19.35%) | 0 (0%) | 1 (3.23%) | 0 (0%) |
| 7.2 Evaluation Design | 31 | **24 (77.42%)** | 13 (41.94%) | 11 (35.48%) | 4 (12.90%) | 2 (6.45%) | 1 (3.23%) |
| 7.3 Outcome Measurement and Analysis | 31 | **24 (77.42%)** | 14 (45.16%) | 10 (32.26%) | 3 (9.68%) | 3 (9.68%) | 1 (3.23%) |
| 7.4 Impact Reporting | 31 | **21 (67.74%)** | 12 (38.71%) | 9 (29.03%) | 5 (16.13%) | 4 (12.90%) | 1 (3.23%) |
| 7.5 Feedback Integration | 31 | 25 (80.65%) | 17 (54.84%) | 8 (25.81%) | 4 (12.90%) | 1 (3.23%) | 1 (3.23%) |
| 7.6 Data-driven advocacy | 31 | **23 (74.2%)** | 10 (32.26%) | 13 (41.94%) | 5 (16.13%) | 2 (6.45%) | 1 (3.23%) |
| 7.7 Reflective practice and continuous improvement | 31 | 30 (96.77%) | 22 (70.97%) | 8 (25.81%) | 0 (0%) | 1 (3.23%) | 0 (0%) |
| 7.8 Systemic Change Impact Evaluation | 31 | **22 (70.97%)** | 9 (29.03%) | 13 (41.94%) | 4 (12.90%) | 4 (12.90%) | 1 (3.23%) |
| 8. Supportive Partnerships and System Change | 31 | 30 (96.77%) | 17 (54.84%) | 13 (41.94%) | 1 (3.23%) | 0 (0%) | 0 (0%) |
| 8.1 Supportive Partnerships and Advocacy | 31 | 29 (93.54%) | 25 (80.65%) | 4 (12.90%) | 1 (3.23%) | 1 (3.23%) | 0 (0%) |
| 8.2 Aggregating Stories and Data to identify systemic issues | 31 | 27 (83.87%) | 15 (48.39%) | 11 (35.48%) | 2 (6.45%) | 3 (9.68%) | 0 (0%) |
| 8.3 Cross-sector collaboration | 31 | 31 (100%) | 21 (67.74%) | 10 (32.26%) | 0 (0%) | 0 (0%) | 0 (0%) |
| 8.4 Policy engagement and influence | 31 | **24 (77.42%)** | 12 (38.71%) | 12 (38.71%) | 7 (22.58%) | 0 (0%) | 0 (0%) |
| 8.5 Local needs assessment and reporting | 31 | 29 (93.54%) | 16 (51.61%) | 13 (41.94%) | 2 (6.45%) | 0 (0%) | 0 (0%) |
| 8.6 Systems-level impact evaluation | 31 | **20 (64.51%)** | 9 (29.03%) | 11 (35.48%) | 5 (16.13%) | 6 (19.35%) | 0 (0%) |
| 8.7 Systemic understanding and contribution | 31 | 25 (80.65%) | 15 (48.39%) | 10 (32.26%) | 5 (16.13%) | 1 (3.23%) | 0 (0%) |
| 9. Integration of Environment and Contextual Factors in Practice | 31 | 27 (83.87%) | 18 (58.06%) | 9 (29.03%) | 4 (12.90%) | 0 (0%) | 0 (0%) |
| 9.1 Environmental health and wellbeing assessment | 31 | 30 (96.77%) | 22 (70.97%) | 8 (25.81%) | 1 (3.23%) | 0 (0%) | 0 (0%) |
| 9.2 Accessibility and environmental analysis | 31 | 29 (93.54%) | 23 (74.19%) | 6 (19.35%) | 2 (6.45%) | 0 (0%) | 0 (0%) |
| 9.3 Natural resource integration and sustainable practice | 31 | 28 (90.32%) | 24 (77.42%) | 4 (12.90%) | 3 (9.68%) | 0 (0%) | 0 (0%) |
| 9.4 Climate-resilient health planning | 31 | **17 (54.84%)** | 12 (38.71%) | 5 (16.13%) | 7 (22.58%) | 6 (19.35%) | 1 (3.23%) |
| 9.5 Environmental health education | 31 | **22** **(70.97%)** | 13 (41.94%) | 9 (29.03%) | 8 (25.81%) | 1 (3.23%) | 0 (0%) |
| 9.6 Sustainable community | 31 | **22 (70.97%)** | 11 (35.48%) | 11 (35.48%) | 6 (19.35%) | 3 (9.68%) | 0 (0%) |

*Round 1 Results are the sum of the participants who rated their agreement as Somewhat Agree (4) or Completely Agree (5)

† Round 1 Results that are <80% have been bolded to denote that consensus was not reached

# Round 1 for International Experts

|  | **N** | **Round 1 Results***† | **N Completely Agree (5)** | **N Somewhat Agree (4)** | **N Neither Agree nor Disagree (3)** | **N Somewhat Disagree (2)** | **N Completely Disagree (1)** |
| --- | --- | --- | --- | --- | --- | --- | --- |
| **Graduate Attributes** |  |  |  |  |  |  |  |
| 1. Ethical Practitioner | 11 | 10 (90.91%) | 9 (81.82%) | 1 (9.09%) | 0 (0%) | 0 (0%) | 1 (9.09%) |
| 1.1 Professional Integrity | 11 | 11 (100%) | 10 (90.91%) | 1 (9.09%) | 0 (0%) | 0 (0%) | 0 (0%) |
| 1.2 Ethical Awareness and Reasoning | 11 | 11 (100%) | 7 (63.64%) | 4 (36.36%) | 0 (0%) | 0 (0%) | 0 (0%) |
| 1.3 Ethical Decision-Making | 11 | 11 (100%) | 9 (81.82%) | 2 (18.18%) | 0 (0%) | 0 (0%) | 0 (0%) |
| 1.4 Legan and Ethical Compliance | 11 | 11 (100%) | 9 (81.82%) | 2 (18.18%) | 0 (0%) | 0 (0%) | 0 (0%) |
| 1.5 Holistic Awareness | 11 | 11 (100%) | 6 (54.55%) | 5 (45.45%) | 0 (0%) | 0 (0%) | 0 (0%) |
| 2. Inclusive and Respectful | 11 | 10 (90.91%) | 10 (90.91%) | 0 (0%) | 0 (0%) | 0 (0%) | 1 (9.09%) |
| 2.1 Cultural Respect | 11 | 11 (100%) | 10 (90.91%) | 1 (9.09%) | 0 (0%) | 0 (0%) | 0 (0%) |
| 2.2 Cultural Awareness | 11 | 11 (100%) | 8 (72.73%) | 3 (27.27%) | 0 (0%) | 0 (0%) | 0 (0%) |
| 2.3 Cultural Humility | 11 | 11 (100%) | 7 (63.64%) | 4 (36.36%) | 0 (0%) | 0 (0%) | 0 (0%) |
| 2.4 Inclusive Mindset | 11 | 11 (100%) | 11 (100%) | 0 (0%) | 0 (0%) | 0 (0%) | 0 (0%) |
| 2.5 Commitment to Equity | 11 | 11 (100%) | 11 (100%) | 0 (0%) | 0 (0%) | 0 (0%) | 0 (0%) |
| 3. Effective Communicator | 11 | 10 (90.91%) | 9 (81.82%) | 1 (9.09%) | 0 (0%) | 0 (0%) | 1 (9.09%) |
| 3.1 Adaptive Communication | 11 | 11 (100%) | 10 (90.91%) | 1 (9.09%) | 0 (0%) | 0 (0%) | 0 (0%) |
| 3.2 Active Listening and Deep Engagement | 11 | 11 (100%) | 10 (90.91%) | 1 (9.09%) | 0 (0%) | 0 (0%) | 0 (0%) |
| 3.3 Empathetic and Person-Centred Communication | 11 | 11 (100%) | 10 (90.91%) | 1 (9.09%) | 0 (0%) | 0 (0%) | 0 (0%) |
| 3.4 Management of Sensitive Conversations | 11 | 10 (90.91%) | 8 (72.73%) | 2 (18.18%) | 1 (9.09%) | 0 (0%) | 0 (0%) |
| 3.5 Using techniques to support action | 11 | 10 (90.91%) | 9 (81.82%) | 1 (9.09%) | 1 (9.09%) | 0 (0%) | 0 (0%) |
| 4. Critical Thinker and Problem Solver | 11 | 10 (90.91%) | 7 (63.64%) | 3 (27.27%) | 0 (0%) | 1 (9.09%) | 0 (0%) |
| 4.1 Analytical and Innovative Thinking | 11 | 11 (100%) | 5 (45.45%) | 6 (54.55%) | 0 (0%) | 0 (0%) | 0 (0%) |
| 4.2 Evidence-based practice and evaluation | 11 | **8 (72.72%)** | 3 (27.27%) | 5 (45.45%) | 3 (27.27%) | 0 (0%) | 0 (0%) |
| 4.3 Adaptive problem solving | 11 | 10 (90.91%) | 7 (63.64%) | 3 (27.27%) | 1 (9.09%) | 0 (0%) | 0 (0%) |
| 4.4 Future-focused strategic planning | 11 | **8 (72.72%)** | 2 (18.18%) | 6 (54.55%) | 3 (27.27%) | 0 (0%) | 0 (0%) |
| 4.5 Resource gap analysis and development | 11 | 9 (81.82%) | 6 (54.55%) | 3 (27.27%) | 2 (18.18%) | 0 (0%) | 0 (0%) |
| 4.6 Inclusive Thinking and Creativity | 11 | 10 (90.91%) | 5 (45.45%) | 5 (45.45%) | 1 (9.09%) | 0 (0%) | 0 (0%) |
| 5. Systemic Change Catalyst | 11 | 9 (81.82%) | 6 (54.55%) | 3 (27.27%) | 1 (9.09%) | 1 (9.09%) | 0 (0%) |
| 5.1 Systems Understanding | 11 | 10 (90.91%) | 9 (81.82%) | 1 (9.09%) | 1 (9.09%) | 0 (0%) | 0 (0%) |
| 5.2 Improvement Mindset | 11 | 10 (90.91%) | 7 (63.64%) | 3 (27.27%) | 1 (9.09%) | 0 (0%) | 0 (0%) |
| 5.3 Collective Action Contribution | 11 | 9 (81.82%) | 8 (72.73%) | 1 (9.09%) | 2 (18.18%) | 0 (0%) | 0 (0%) |
| 5.4 Intentional and strategic | 11 | 10 (90.91%) | 3 (27.27%) | 7 (63.64%) | 1 (9.09%) | 0 (0%) | 0 (0%) |
| 5.5 Resilience and persistence | 11 | 9 (81.82%) | 5 (45.45%) | 4 (36.36%) | 2 (18.18%) | 0 (0%) | 0 (0%) |
| 6. Community Empowerment and Collaboration | 11 | 10 (90.91%) | 9 (81.82%) | 1 (9.09%) | 1 (9.09%) | 0 (0%) | 0 (0%) |
| 6.1 Health Equity Approach | 11 | 11 (100%) | 11 (100%) | 0 (0%) | 0 (0%) | 0 (0%) | 0 (0%) |
| 6.2 Community Empowerment and capacity building | 11 | 9 (81.82%) | 8 (72.73%) | 1 (9.09%) | 2 (18.18%) | 0 (0%) | 0 (0%) |
| 6.3 Collaborative partnership development | 11 | 11 (100%) | 10 (90.91%) | 1 (9.09%) | 0 (0%) | 0 (0%) | 0 (0%) |
| 6.4 Interprofessional collaboration | 11 | 11 (100%) | 8 (72.73%) | 3 (27.27%) | 0 (0%) | 0 (0%) | 0 (0%) |
| 6.5 Facilitation of collaborative decision-making | 11 | 9 (81.82%) | 3 (27.27%) | 6 (54.55%) | 1 (9.09%) | 1 (9.09%) | 0 (0%) |
| 6.6 Supporting positive change | 11 | 10 (90.91%) | 6 (54.55%) | 4 (36.36%) | 1 (9.09%) | 0 (0%) | 0 (0%) |
| 7. Reflective and lifelong learner | 11 | 10 (90.91%) | 7 (63.64%) | 3 (27.27%) | 1 (9.09%) | 0 (0%) | 0 (0%) |
| 7.1 Evidence-based practice | 11 | 9 (81.82%) | 6 (54.55%) | 3 (27.27%) | 2 (18.18%) | 0 (0%) | 0 (0%) |
| 7.2 Reflective practice | 11 | 11 (100%) | 9 (81.82%) | 2 (18.18%) | 0 (0%) | 0 (0%) | 0 (0%) |
| 7.3 Continuous professional development | 11 | 11 (100%) | 6 (54.55%) | 5 (45.45%) | 0 (0%) | 0 (0%) | 0 (0%) |
| 7.4 Professional supervision, peer learning, and mentorship | 11 | 11 (100%) | 8 (72.73%) | 3 (27.27%) | 0 (0%) | 0 (0%) | 0 (0%) |
| 7.5 Self care and collective resilience | 11 | 11 (100%) | 8 (72.73%) | 3 (27.27%) | 0 (0%) | 0 (0%) | 0 (0%) |
|  |  |  |  |  |  |  |  |
| **Core Competencies** |  |  |  |  |  |  |  |
| 1. Working across Australian Health and Social Systems | 11 | 11 (100%) | 6 (54.55%) | 5 (45.45%) | 0 (0%) | 0 (0%) | 0 (0%) |
| 1.1 Multi-level system understanding | 11 | 10 (90.91%) | 5 (45.45%) | 5 (45.45%) | 1 (9.09%) | 0 (0%) | 0 (0%) |
| 1.2 Understand and respond to local and regional needs | 11 | 11 (100%) | 5 (45.45%) | 6 (54.55%) | 0 (0%) | 0 (0%) | 0 (0%) |
| 1.3 Cross-sectoral integration | 11 | 11 (100%) | 8 (72.73%) | 3 (27.27%) | 0 (0%) | 0 (0%) | 0 (0%) |
| 1.4 Rural and remote adaptability | 11 | 11 (100%) | 6 (54.55%) | 5 (45.45%) | 0 (0%) | 0 (0%) | 0 (0%) |
| 1.5 Policy alignment and strategic integration | 11 | **8 (72.72%)** | 3 (27.27%) | 5 (45.45%) | 3 (27.27%) | 0 (0%) | 0 (0%) |
| 1.6 Funding and resource navigation | 11 | 9 (81.82%) | 4 (36.36%) | 5 (45.45%) | 2 (18.18%) | 0 (0%) | 0 (0%) |
| 1.7 Integration with existing programs and schemes | 11 | 9 (81.82%) | 4 (36.36%) | 5 (45.45%) | 2 (18.18%) | 0 (0%) | 0 (0%) |
| 1.8 Digital health integration | 11 | 9 (81.82%) | 9 (81.82%) | 0 (0%) | 2 (18.18%) | 0 (0%) | 0 (0%) |
| 2. Working with people on what matters | 11 | 11 (100%) | 11 (100%) | 0 (0%) | 0 (0%) | 0 (0%) | 0 (0%) |
| 2.1 Empathetic and adaptive communication | 11 | 11 (100%) | 11 (100%) | 0 (0%) | 0 (0%) | 0 (0%) | 0 (0%) |
| 2.2 Inclusive and context-sensitive collaboration | 11 | 11 (100%) | 9 (81.82%) | 2 (18.18%) | 0 (0%) | 0 (0%) | 0 (0%) |
| 2.3 Comprehensive strengths and needs assessment | 11 | 11 (100%) | 10 (90.91%) | 1 (9.09%) | 0 (0%) | 0 (0%) | 0 (0%) |
| 2.4 Collaborative goal setting and social prescription co-design | 11 | 11 (100%) | 9 (81.82%) | 2 (18.18%) | 0 (0%) | 0 (0%) | 0 (0%) |
| 2.5 Holistic health enablement | 11 | 11 (100%) | 8 (72.73%) | 3 (27.27%) | 0 (0%) | 0 (0%) | 0 (0%) |
| 2.6 Adaptable planning | 11 | 10 (90.91%) | 8 (72.73%) | 2 (18.18%) | 1 (9.09%) | 0 (0%) | 0 (0%) |
| 2.7 Interprofessional Collaboration | 11 | 11 (100%) | 7 (63.64%) | 4 (36.36%) | 0 (0%) | 0 (0%) | 0 (0%) |
| 3. Resource Navigation and Management | 11 | 11 (100%) | 7 (63.64%) | 4 (36.36%) | 0 (0%) | 0 (0%) | 0 (0%) |
| 3.1 Community Resource Mapping and Knowledge Management | 11 | 10 (90.91%) | 7 (63.64%) | 3 (27.27%) | 1 (9.09%) | 0 (0%) | 0 (0%) |
| 3.2 Sustainable Resource Utilisation and Matching | 11 | 10 (90.91%) | 4 (36.36%) | 6 (54.55%) | 0 (0%) | 1 (9.09%) | 0 (0%) |
| 3.3 Resource Optimisation and Development | 11 | 9 (81.82%) | 5 (45.45%) | 4 (36.36%) | 1 (9.09%) | 1 (9.09%) | 0 (0%) |
| 3.4 Systems Navigation | 11 | 10 (90.91%) | 6 (54.55%) | 4 (36.36%) | 1 (9.09%) | 0 (0%) | 0 (0%) |
| 3.5 Barrier Identification and Mitigation | 11 | 10 (90.91%) | 7 (63.64%) | 3 (27.27%) | 1 (9.09%) | 0 (0%) | 0 (0%) |
| 3.6 Addressing the Digital Divide | 11 | **8 (72.72%)** | 5 (45.45%) | 3 (27.27%) | 2 (18.18%) | 1 (9.09%) | 0 (0%) |
| 3.7 Collaborative Problem-Solving | 11 | 11 (100%) | 9 (81.82%) | 2 (18.18%) | 0 (0%) | 0 (0%) | 0 (0%) |
| 3.8 Critical Analysis and Innovation | 11 | 9 (81.82%) | 3 (27.27%) | 6 (54.55%) | 2 (18.18%) | 0 (0%) | 0 (0%) |
| 4. Community Development and Social Capital Building | 11 | 10 (90.91%) | 7 (63.64%) | 3 (27.27%) | 1 (9.09%) | 0 (0%) | 0 (0%) |
| 4.1 Community Engagement and Empowerment | 11 | 11 (100%) | 4 (36.36%) | 7 (63.64%) | 0 (0%) | 0 (0%) | 0 (0%) |
| 4.2 Social Network and capital building | 11 | 10 (90.91%) | 5 (45.45%) | 5 (45.45%) | 1 (9.09%) | 0 (0%) | 0 (0%) |
| 4.3 Inclusive practice and cross-cultural bridging | 11 | 10 (90.91%) | 7 (63.64%) | 3 (27.27%) | 1 (9.09%) | 0 (0%) | 0 (0%) |
| 4.4 Supporting community-led initiatives | 11 | 10 (90.91%) | 5 (45.45%) | 5 (45.45%) | 1 (9.09%) | 0 (0%) | 0 (0%) |
| 4.5 Collaborative partnership development | 11 | 10 (90.91%) | 6 (54.55%) | 4 (36.36%) | 1 (9.09%) | 0 (0%) | 0 (0%) |
| 4.6 Interest balancing and conflict resolution | 11 | 9 (81.82%) | 4 (36.36%) | 5 (45.45%) | 2 (18.18%) | 0 (0%) | 0 (0%) |
| 4.7 Sustainable community capacity building | 11 | 9 (81.82%) | 4 (36.36%) | 5 (45.45%) | 1 (9.09%) | 1 (9.09%) | 0 (0%) |
| 4.8 Community-led framework implementation | 11 | 10 (90.91%) | 5 (45.45%) | 5 (45.45%) | 1 (9.09%) | 0 (0%) | 0 (0%) |
| 5. Culturally Safe and Inclusive Practice | 11 | 11 (100%) | 10 (90.91%) | 1 (9.09%) | 0 (0%) | 0 (0%) | 0 (0%) |
| 5.1 Cultural competence development | 11 | 11 (100%) | 10 (90.91%) | 1 (9.09%) | 0 (0%) | 0 (0%) | 0 (0%) |
| 5.2 Cultural safety implementation | 11 | 11 (100%) | 10 (90.91%) | 1 (9.09%) | 0 (0%) | 0 (0%) | 0 (0%) |
| 5.3 Inclusive Service delivery | 11 | 11 (100%) | 10 (90.91%) | 1 (9.09%) | 0 (0%) | 0 (0%) | 0 (0%) |
| 5.4 Transcultural connectivity and adaptability | 11 | 11 (100%) | 9 (81.82%) | 2 (18.18%) | 0 (0%) | 0 (0%) | 0 (0%) |
| 6. Safe and Effective Practice | 11 | 11 (100%) | 10 (90.91%) | 1 (9.09%) | 0 (0%) | 0 (0%) | 0 (0%) |
| 6.1 Comprehensive risk management | 11 | 10 (90.91%) | 9 (81.82%) | 1 (9.09%) | 1 (9.09%) | 0 (0%) | 0 (0%) |
| 6.2 Navigation of complex situations | 11 | 11 (100%) | 8 (72.73%) | 3 (27.27%) | 0 (0%) | 0 (0%) | 0 (0%) |
| 6.3 Ethical decision-making | 11 | 10 (90.91%) | 8 (72.73%) | 2 (18.18%) | 1 (9.09%) | 0 (0%) | 0 (0%) |
| 6.4 Legal compliance and documentation | 11 | 10 (90.91%) | 9 (81.82%) | 1 (9.09%) | 1 (9.09%) | 0 (0%) | 0 (0%) |
| 6.5 Managing crises | 11 | 9 (81.82%) | 7 (63.64%) | 2 (18.18%) | 1 (9.09%) | 1 (9.09%) | 0 (0%) |
| 6.6 Adaptive practice and safety enhancement | 11 | 11 (100%) | 7 (63.64%) | 4 (36.36%) | 0 (0%) | 0 (0%) | 0 (0%) |
| 6.7 Conflict management | 11 | 9 (81.82%) | 4 (36.36%) | 5 (45.45%) | 1 (9.09%) | 1 (9.09%) | 0 (0%) |
| 7. Data Management and Evaluation | 11 | 9 (81.82%) | 6 (54.55%) | 3 (27.27%) | 2 (18.18%) | 0 (0%) | 0 (0%) |
| 7.1 Ethical and Legal Data Management | 11 | 9 (81.82%) | 8 (72.73%) | 1 (9.09%) | 2 (18.18%) | 0 (0%) | 0 (0%) |
| 7.2 Evaluation Design | 11 | **8 (72.72%)** | 3 (27.27%) | 5 (45.45%) | 2 (18.18%) | 1 (9.09%) | 0 (0%) |
| 7.3 Outcome Measurement and Analysis | 11 | **8 (72.72%)** | 4 (36.36%) | 4 (36.36%) | 2 (18.18%) | 1 (9.09%) | 0 (0%) |
| 7.4 Impact Reporting | 11 | **8 (72.72%)** | 3 (27.27%) | 5 (45.45%) | 2 (18.18%) | 1 (9.09%) | 0 (0%) |
| 7.5 Feedback Integration | 11 | **8 (72.72%)** | 4 (36.36%) | 4 (36.36%) | 3 (27.27%) | 0 (0%) | 0 (0%) |
| 7.6 Data-driven advocacy | 11 | 9 (81.82%) | 4 (36.36%) | 5 (45.45%) | 2 (18.18%) | 0 (0%) | 0 (0%) |
| 7.7 Reflective practice and continuous improvement | 11 | 9 (81.82%) | 8 (72.73%) | 1 (9.09%) | 2 (18.18%) | 0 (0%) | 0 (0%) |
| 7.8 Systemic Change Impact Evaluation | 11 | **7 (63.63%)** | 2 (18.18%) | 5 (45.45%) | 3 (27.27%) | 1 (9.09%) | 0 (0%) |
| 8. Supportive Partnerships and System Change | 11 | 9 (81.82%) | 4 (36.36%) | 5 (45.45%) | 1 (9.09%) | 1 (9.09%) | 0 (0%) |
| 8.1 Supportive Partnerships and Advocacy | 11 | 10 (90.91%) | 8 (72.73%) | 2 (18.18%) | 1 (9.09%) | 0 (0%) | 0 (0%) |
| 8.2 Aggregating Stories and Data to identify systemic issues | 11 | 10 (90.91%) | 3 (27.27%) | 7 (63.64%) | 1 (9.09%) | 0 (0%) | 0 (0%) |
| 8.3 Cross-sector collaboration | 11 | 10 (90.91%) | 6 (54.55%) | 4 (36.36%) | 1 (9.09%) | 0 (0%) | 0 (0%) |
| 8.4 Policy engagement and influence | 11 | **8 (72.72%)** | 3 (27.27%) | 5 (45.45%) | 2 (18.18%) | 1 (9.09%) | 0 (0%) |
| 8.5 Local needs assessment and reporting | 11 | **8 (72.72%)** | 3 (27.27%) | 5 (45.45%) | 3 (27.27%) | 0 (0%) | 0 (0%) |
| 8.6 Systems-level impact evaluation | 11 | **8 (72.72%)** | 2 (18.18%) | 6 (54.55%) | 1 (9.09%) | 2 (18.18%) | 0 (0%) |
| 8.7 Systemic understanding and contribution | 11 | 10 (90.91%) | 3 (27.27%) | 7 (63.64%) | 1 (9.09%) | 0 (0%) | 0 (0%) |
| 9. Integration of Environment and Contextual Factors in Practice | 11 | 10 (90.91%) | 6 (54.55%) | 4 (36.36%) | 1 (9.09%) | 0 (0%) | 0 (0%) |
| 9.1 Environmental health and wellbeing assessment | 11 | 11 (100%) | 7 (63.64%) | 4 (36.36%) | 0 (0%) | 0 (0%) | 0 (0%) |
| 9.2 Accessibility and environmental analysis | 11 | 11 (100%) | 9 (81.82%) | 2 (18.18%) | 0 (0%) | 0 (0%) | 0 (0%) |
| 9.3 Natural resource integration and sustainable practice | 11 | 9 (81.82%) | 5 (45.45%) | 4 (36.36%) | 2 (18.18%) | 0 (0%) | 0 (0%) |
| 9.4 Climate-resilient health planning | 11 | **8 (72.72%)** | 4 (36.36%) | 4 (36.36%) | 3 (27.27%) | 0 (0%) | 0 (0%) |
| 9.5 Environmental health education | 11 | 9 (81.82%) | 6 (54.55%) | 3 (27.27%) | 2 (18.18%) | 0 (0%) | 0 (0%) |
| 9.6 Sustainable community | 11 | **8 (72.72%)** | 4 (36.36%) | 4 (36.36%) | 2 (18.18%) | 1 (9.09%) | 0 (0%) |

# Round 2 Revised Items

| **Original** | | **Revised** | |
| --- | --- | --- | --- |
| **Graduate Attributes** |  |  |  |
| *4.2 Evidence-based practice and evaluation* | Integrate current research and best practices in social care and community health. Critically evaluate the effectiveness of social prescribing interventions, using data to inform ongoing practice improvements | *4.2 Evidence-Informed Practice* | Link workers use established evidence-informed approaches in their practice and contribute to evaluation activities through accurate data collection and documentation |
| *4.4 Future-focused strategic planning* | Co-design long-term strategies for community health improvements, anticipating future challenges and opportunities, and considering impacts on communities and environmental contexts | *4.4 Supporting Community-Focused Initiatives* | Collaborate with team members and community stakeholders to contribute insights from day-to-day practice that inform organisational or regional strategies for health improvements. Focus on identifying immediate opportunities and barriers to enhance community health within the context of current resources and priorities. |
| *5.5 Resilience and persistence* | Demonstrate tenacity in pursuing systemic changes, maintaining motivation and inspiring others in the face of bureaucratic or institutional resistance | *5.5 Resilience and Adaptability* | Maintain a positive and solution-focused approach when navigating challenges within service delivery, demonstrating persistence in addressing individual and community needs. Support clients and colleagues by fostering motivation and adaptability, even in the face of organisational or systemic barriers. |
| **Core Competencies** |  |  |  |
| *1.6 Funding and resource navigation* | Navigate and leverage diverse Australian health and social care funding mechanisms to support social prescribing initiatives. This includes understanding and utilising Medicare item numbers, state funding, the National Disability Insurance Scheme, My Aged Care, Domestic and Family Violence Fund, Disaster Recovery Allowance, and community health grants | *1.6 Funding and resource navigation* | Link workers maintain comprehensive knowledge of community assets, government funding schemes, and local service landscapes. This includes understanding opportunities available from their local government, as well as an appreciation of state and Commonwealth funded services and supports. They support clients to access and navigate available resources while systematically documenting gaps and barriers encountered. They understand how different funding schemes operate and help connect clients with appropriate support options, maintaining current knowledge of eligibility criteria and access pathways. |
| *7.4 Impact Reporting* | Translate complex data into clear, compelling reports for diverse audiences, articulate the value and outcomes of social prescribing to partners, policymakers, and the community | *Evidence Collection for System Impact* | Link workers systematically document client journeys, experiences, and outcomes using standardised tools and approaches. They capture patterns in social and practical needs, record successful interventions and challenges, and gather client feedback about service experiences. They contribute valuable ground-level insights about service gaps and solutions while maintaining accurate records of community needs and responses |
| *7.8 Systemic Change Impact Evaluation* | Design and implement evaluation frameworks that assess the impact of link workers' efforts in driving systemic change. Measure and report on how identified local needs and systemic barriers are being addressed at larger governmental and policy levels |  |  |
| *1.5 Policy Alignment and Strategic Integration* | Demonstrate a comprehensive understanding of key national health policies, such as the National Preventive Health Strategy and Primary Health Care 10 Year Plan, and strategically align social prescribing practices within these frameworks to enhance their impact and sustainability | *Awareness and Communication of Policy Impacts* | Link workers stay informed about how policies and funding mechanisms affect client access and outcomes in their community. They identify and document barriers, enablers, and patterns observed in their work, passing on these insights to supervisors or relevant stakeholders to support service improvements and planning. |
| *8.4 Policy Engagement and Influence* | Stay informed about policy changes affecting social prescribing. Contribute to policy discussions based on front-line experiences. Engage proactively with policymakers and commissioning bodies to advocate for changes that address systemic barriers and promote holistic health outcomes |  |  |
| *4.6 Interest Balancing and Conflict Resolution* | Navigate and harmonise diverse community interests, facilitating inclusive discussions to reach collaborative solutions that respect different needs and viewpoints | *4.6 Navigating Complex Situations* | Link workers understand how to navigate challenging interpersonal and systemic situations while maintaining clear role boundaries. They employ basic de-escalation techniques when appropriate, recognise when to seek support or refer to qualified services, and follow organisational procedures for addressing conflicts. They contribute to positive relationships while clearly understanding the limits of their role in resolving complex situations |
| *9.4. Climate-Resilient Health Planning* | Consider climate change impacts when designing long-term health improvement strategies. Develop social prescribing approaches that address Australia's specific climate challenges, including bushfire preparedness, drought resilience, and extreme heat adaptation. Integrate climate mitigation and adaptation principles into practices | *Environmental Health Awareness* | Link workers understand how environmental factors affect community wellbeing and can connect clients with relevant local environmental programs and green spaces. They maintain awareness of environmental health impacts while working within their scope to support access to environmentally supportive activities and resources |
| *9.5. Environmental Health Education* | Facilitate learning opportunities that enhance understanding of the connections between personal health, community wellbeing, and environmental stewardship. |  |  |
| *9.6. Sustainable Community Initiative Support* | Guide community groups in initiating and implementing environmentally sustainable projects that address local health needs and enhance community wellbeing |  |  |
| **New Skills** |  |  |  |
| *Trust and Relationship Building* | Link workers develop and maintain trusting professional relationships with clients, communities, and service partners. They demonstrate trauma-informed practice, create safe spaces for engagement, work at the client's pace, and foster connections while maintaining appropriate boundaries. They build sustainable relationships that support ongoing engagement and facilitate connections between community members and services. | | |
| *Scope Recognition and Referral Navigation* | Link workers understand the boundaries of social prescribing and recognise when clients need additional or alternative support. They maintain knowledge of appropriate referral pathways, particularly for clinical services, and can effectively communicate these boundaries to clients while maintaining supportive relationships. They work collaboratively with other services to enhance client wellbeing within clear professional parameters. | | |
| *Enterprising and Adaptive Practice* | Link workers creatively utilise available resources and adapt approaches to local contexts and constraints. They demonstrate resilience and innovation in resource-limited settings, identify opportunities for service enhancement, and develop sustainable solutions using existing community assets. They maintain effectiveness while working within varying resource environments and organisational contexts. | | |
| *Digital Literacy and Inclusive Engagement* | Link workers effectively use digital tools while ensuring inclusive practice for those with varying technological access and comfort. They adapt communication methods for different needs, maintain alternative engagement pathways, and support digital inclusion while recognising and addressing technological barriers. They leverage technology appropriately to enhance service delivery and reach underserved populations | | |

# Australian Educational Standards for Social Prescribing Link Workers

## Graduate Attributes

### 1. Ethical

*Social prescribing link workers demonstrate a strong ethical foundation, supporting autonomy, beneficence, and displaying principled decision-making in complex scenarios while considering professional responsibilities and applying holistic and respectful ways of thinking.*

Attribute Indicators:

1. **Professional Integrity**: Uphold and promote ethical standards in all aspects of practice, including confidentiality, informed consent, and respect for autonomy.
2. **Ethical Awareness and Reasoning**: Demonstrate advanced capabilities in recognising and analysing ethical dimensions of social prescribing scenarios.
3. **Ethical Decision-Making**: Apply ethical frameworks to resolve complex dilemmas, considering multiple partner perspectives.
4. **Legal and Ethical Compliance**: Understand and adhere to legal and professional ethical guidelines relevant to social prescribing.
5. **Holistic Awareness**: Demonstrate an understanding of the interconnectedness between individuals, communities, and the natural world. Make ethically sound decisions in social prescribing that respect and promote environmental balance, recognising the equal importance of all beings in maintaining ecosystem health and respecting that contribution.

### 2. Inclusive and Respectful

*Social prescribing link workers embody a deep appreciation for cultural diversity, demonstrating attitudes and awareness that foster inclusive environments and culturally sensitive approaches.*

Attribute Indicators:

1. **Cultural Respect**: Demonstrate commitment to respecting people’s traditional knowledge, customs, heritage, wisdom, and cultural beliefs and practices, with particular regard to Aboriginal and Torres Strait Islander peoples.
2. **Cultural Awareness**: Demonstrate an understanding of diverse cultural perspectives and their impact on health and wellbeing.
3. **Cultural Humility**: Engage in ongoing self-reflection about personal cultural biases and their potential impact on practice.
4. **Inclusive Mindset**: Embrace and value diversity in all its forms, respecting and valuing diverse beliefs, histories, traits, and experiences, including but not limited to culture, ethnicity, race, disability, age, gender, sexuality, socioeconomic status, and religion, fostering an inclusive and supportive environment for all.
5. **Commitment to Equity**: Demonstrate a dedication to promoting equitable access to health and social care services, community supports, and resources, with the goal of achieving improved health and wellbeing outcomes for all individuals and communities.

### 3. Effective Communicator

*Social prescribing link workers display excellence in adaptive, person-centred communication, utilising a range of techniques to engage effectively with diverse individuals and groups in various contexts.*

Attribute Indicators:

1. **Adaptive Communication**: Demonstrate proficiency in adapting communication style and methods to suit individual needs that are respectful and culturally appropriate, including employing a range of questioning techniques to gather accurate information and using various communication tools and technologies, including digital platforms and telehealth services, to ensure inclusive engagement
2. **Active Listening and Deep Engagement**: Exhibit excellence in creating a supportive environment where individuals feel valued and understood through demonstrating attentive listening, using appropriate body language and non-verbal cues, and applying reflective communication techniques to ensure comprehensive understanding of perspectives and desires
3. **Empathetic and Person-Centred Communication**: Show ability to recognise and validate individuals' feelings, thoughts, and behaviours as normal responses to their circumstances. Use reflective techniques to help individuals explore their unique narratives, reframe challenges, and identify sources of resilience and positive emotion
4. **Management of Sensitive Conversations**: Display confidence and sensitivity in navigating difficult topics while maintaining respect and support and discussing complex health and social issues, including referrals to appropriate services and community activities
5. **Using techniques to support action**: Employ evidence-based communication strategies that support individuals to make and maintain sustainable changes that matter to them and empower them to take control of their wellbeing (e.g., motivational interviewing, coaching skills)

### 4. Critical Thinker and Problem Solver

*Social prescribing link workers apply analytical and creative thinking to address complex health and social issues, developing innovative, evidence-based solutions that consider long-term impacts and sustainability.*

Attribute Indicators:

1. **Analytical and Innovative Thinking**: Demonstrate ability to analyse complex social and health issues, identifying root causes and potential solutions. Apply creative thinking to help individuals and communities reframe challenges, discover new meanings, and envision positive future narratives
2. **Evidence**-Informed Practice: Link workers use established evidence-informed approaches in their practice and contribute to evaluation activities through accurate data collection and documentation
3. **Adaptive Problem Solving**: Show flexibility in adapting to new evidence, community needs, and technological advancements and demonstrate skill in navigating and harmonising diverse community interests and priorities, facilitating inclusive discussions to reach collaborative solutions
4. **Supporting Community-Focused Initiatives**: Collaborate with team members and community stakeholders to contribute insights from day-to-day practice that inform organisational or regional strategies for health improvements. Focus on identifying immediate opportunities and barriers to enhance community health within the context of current resources and priorities.
5. **Resource Gap Analysis and Development**: Identify gaps in available resources and services and employ creative problem-solving to address immediate needs and advocate for the development of new resources to meet identified community requirements
6. **Inclusive Thinking and Creativity**: Consider the ethical, cultural, and practical implications of new approaches and innovations in social prescribing and maximise inclusion and minimise inadvertently disadvantaging any group

### 5. Systemic Change Catalyst

*Social prescribing link workers demonstrate an understanding of the broader health and social care systems and recognise the need for systemic transformation to address health inequities and social determinants of wellbeing. They identify opportunities for improvement within their scope of practice and contribute to positive change through collaborative efforts and evidence-based advocacy*

Attribute Indicators:

1. **Systems Understanding:** Demonstrate a basic understanding of the broader health and social care systems, including their strengths and limitations in addressing community needs
2. **Improvement Mindset:** Approach individual cases and community issues with an awareness of how they might inform potential improvements in service delivery or community support
3. **Collective Action Contribution**: Participate in team or organisational efforts aimed at enhancing service quality and addressing systemic challenges within the scope of social prescribing
4. **Intentional and Strategic:** Recognise ethical considerations in advocating for change, balancing immediate needs with long-term goals while maintaining professional integrity
5. **Resilience and Adaptability:** Maintain a positive and solution-focused approach when navigating challenges within service delivery, demonstrating persistence in addressing individual and community needs. Support clients and colleagues by fostering motivation and adaptability, even in the face of organisational or systemic barriers.

### 6. Community Empowerment and Collaboration

*Social prescribing link workers champion community empowerment, facilitate collaborative initiatives that enhance collective wellbeing, foster partnerships, and advocate for systemic changes to improve health equity. They demonstrate leadership in guiding community initiatives and facilitating collaborative decision-making processes that respect diverse perspectives and promote shared goals in health and wellbeing.*

Attribute Indicators:

1. **Health Equity Approach**: Demonstrate understanding of social and structural determinants of health and their impact on individuals, marginalised groups, and communities and apply knowledge of factors such as housing, income, education, culture, social support, and environmental conditions to work towards equitable health opportunities for all
2. **Community Empowerment and Capacity Building**: Embrace principles of community-led development and capacity building. Facilitate and support community members and partners in identifying and achieving shared health and wellbeing goals, enhancing their skills, knowledge, and confidence through collaborative learning processes.
3. **Collaborative Partnership Development**: Foster and maintain robust partnerships among diverse community members, including local organisations, health services, businesses, and community groups and negotiate, coordinate, and manage relationships to achieve shared community health and wellbeing objectives
4. **Interprofessional Collaboration**: Demonstrate proficiency in working across various health, social care, and community sectors. Actively engage in interprofessional teams, recognising and respecting the roles and expertise of different professionals. Facilitate effective communication and collaboration between diverse professional groups to enhance the comprehensiveness and effectiveness of social prescribing practices.
5. **Facilitation of Collaborative Decision-Making**: Facilitate inclusive discussions and guide communities towards collaborative solutions. Balance diverse community interests and priorities, ensuring all voices are heard and respected
6. **Supporting Positive Change:** Demonstrate a commitment to supporting individuals and communities, using data and insights, to address systemic barriers to health and wellbeing

### 7. Reflective and Lifelong Learner

*Social prescribing link workers engage in continuous self-improvement and professional development, regularly reflecting on their practice and staying abreast of current research and best practices in social care and community health.*

Attribute Indicators:

1. **Evidence-Based Practice**: Commit to basing work on the latest research and best practices in social care and community health and continuously evaluate and adapt approaches based on feedback, outcomes, and professional insights
2. **Reflective Practice**: Engage in ongoing self-assessment and learning from experiences, consider the impact of actions and decisions on personal competencies and people's outcomes, and apply reflective thinking to understand and navigate complex ethical dilemmas
3. **Continuous Professional Development**: Demonstrate commitment to ongoing learning and skill enhancement in practical, legal, and ethical dimensions of social prescribing and actively pursue opportunities to master current research and understand comprehensive legal responsibilities
4. **Professional Supervision, Peer Learning, and Mentorship:** Participate actively in regular, structured supervision sessions with qualified supervisors. Engage in peer-to-peer learning opportunities and contribute to the professional community through sharing insights and best practices. Seek mentorship to support personal growth and, as experience develops, engage in mentoring roles to support the development of other professionals in the field
5. **Self-care and Collective Resilience:** Develop and maintain personal and organisational strategies for managing stress and preventing burnout, while fostering a supportive work culture that prioritises employee wellbeing in a challenging work environment

## Core Competencies

### 1. Working Across Australian Health and Social Systems

*Social prescribing link workers demonstrate a comprehensive understanding of Australia's complex health and social systems, effectively navigating its multi-tiered structure to integrate social prescribing within existing health and social care frameworks.*

Associated Skills:

1. **Multi-level System Understanding**: Demonstrate thorough knowledge of Australia's federal, state/territory, and local health and social systems and structures, including Medicare, My Aged Care, the NDIS, the Pharmaceutical Benefits Scheme, and state-based health services.
2. **Understand and Respond to Local and Regional Needs**: Effectively use documents produced by Primary Health Networks, local governments and other needs assessments and plans to ensure social prescribing initiatives align with regional health priorities and contribute to population health planning.
3. **Cross-sectoral Integration**: Navigate and foster partnerships across public, private, and community health sectors, integrating social prescribing within Australia's unique healthcare landscape.
4. **Rural and Remote Adaptability**: Develop and implement social prescribing strategies tailored to the unique challenges and opportunities of rural and remote Australian communities including strategies for engaging hard-to-reach or underserviced communities.
5. **Funding and Resource Navigation**: Link workers maintain comprehensive knowledge of community assets, government funding schemes, and local service landscapes. This includes understanding opportunities available from their local government, as well as an appreciation of state and Commonwealth funded services and supports. They support clients to access and navigate available resources while systematically documenting gaps and barriers encountered. They understand how different funding schemes operate and help connect clients with appropriate support options, maintaining current knowledge of eligibility criteria and access pathways.
6. **Integration with Existing Programs and Schemes**: Skilfully integrate social prescribing within funding schemes such as Australia's mental health initiatives, My Aged Care, the NDIS, skills building programs, and other specialised programs. Demonstrate proficiency in understanding and utilising schemes such as Medicare Better Access, state-based mental health programs, and other relevant specialised health and social care initiatives.
7. **Digital Health Integration**: Demonstrate proficiency in navigating and utilising digital health platforms and technologies relevant to social prescribing in Australia. This includes telehealth services, My Health Record, health apps, and online health information resources. Understand the benefits and limitations of digital health tools in the context of social prescribing and assist clients in accessing and using these technologies effectively

### 2. Working with People on What Matters

*Social prescribing link workers demonstrate expertise in engaging individuals in a holistic, strengths-based manner, collaboratively assessing needs and co-creating tailored social prescriptions that align with personal goals and values.*

Associated Skills:

- 1. **Empathetic and Adaptive Communication**: Create a supportive and empowering environment where individuals feel genuinely heard and valued. Apply active listening, empathy, and adaptive questioning techniques to gather accurate information and facilitate open sharing of personal stories, challenges, and aspirations. Demonstrate confidence in managing sensitive conversations, ensuring respectful and supportive engagement
  2. **Inclusive and Context-Sensitive Collaboration:** Design and implement social prescribing programs in partnership with diverse community members. Ensure accessibility and relevance for all, considering factors such as age, disability, cultural background, and life experiences. Adjust approaches to provide inclusive, appropriate support, with particular consideration for marginalised groups.
  3. **Comprehensive Strengths and Needs Assessment**: Undertake a holistic stocktake of individuals' strengths, needs, and circumstances across physical, emotional, social, and cultural dimensions. Explore personal narratives to uncover sources of meaning, positive experiences, and opportunities for growth and accomplishment
  4. **Collaborative Goal Setting and Social Prescription Co-Design:** Facilitate collaborative goal setting based on the person's values, needs, and deeper aspirations. Co-create tailored social prescriptions and personalised action plans that are realistic, inspiring, and aligned with individual capabilities and preferences, fostering engagement and a sense of purpose. Ensure active participation of and leadership by individuals in the decision-making process
  5. **Holistic Health Enablement**: Support individuals in navigating the social and structural determinants of health that influence their available resources and behaviours. Guide them in understanding and addressing both personal and systemic factors affecting their health and wellbeing. Assist in developing strategies to overcome individual barriers while recognising larger systemic issues that may need to be addressed at a population level.
  6. **Adaptable Planning**: Link people with options and opportunities that are flexible and adaptable over time and regularly review and refine action plans to ensure ongoing alignment with evolving goals and circumstances
  7. **Interprofessional Collaboration**: Actively engage in interprofessional teams, recognising and respecting the roles and expertise of different health and social care professionals. Facilitate effective communication and collaboration between diverse professional groups to enhance the comprehensiveness and effectiveness of social prescribing practices. Navigate potential challenges in interprofessional settings and advocate for the value of social prescribing within multidisciplinary care approaches
  8. **Trust and Relationship Building:** Link workers develop and maintain trusting professional relationships with clients, communities, and service partners. They demonstrate trauma-informed practice, create safe spaces for engagement, work at the client's pace, and foster connections while maintaining appropriate boundaries. They build sustainable relationships that support ongoing engagement and facilitate connections between community members and services.

### 3. Resource Navigation and Management

*Social prescribing link workers expertly identify, map, and utilise community resources. They guide individuals through health and social care systems, match resources to needs, and address access barriers, whilst emphasising sustainable and equitable resource use.*

Associated Skills:

1. **Community Resource Mapping and Knowledge Management:** Conduct and maintain comprehensive mapping of local and national resources across various sectors at local, state, and national levels. Continuously update understanding of available services, programs, and opportunities, including those in the natural environment.
2. **Sustainable Resource Utilisation and Matching**: Develop strategies for efficient, equitable, and environmentally friendly use of community resources. Align available resources with individual client needs, preferences, and circumstances to create comprehensive support networks.
3. **Resource Optimisation and Development**: Work alongside individuals and communities to maximise utilisation of available resources, including identifying and leveraging unconventional or underutilised assets. Conduct gap analyses to identify resource shortfalls, creatively problem-solve to meet immediate needs, and advocate for the development of new resources. Co-design and implement community health initiatives that effectively align available and potential resources with individual and community needs.
4. **Systems Navigation**: Guide individuals through complex health and social care systems, understanding referral pathways and eligibility criteria. Assist in accessing and navigating various services and support mechanisms.
5. **Barrier Identification and Mitigation**: Identify and address barriers to resource access, including informational, physical, transport, financial, and language obstacles.
6. **Addressing the Digital Divide:** Enhance individuals' ability to access and navigate online government services, digital health resources, and telehealth platforms. Support people in developing digital skills to effectively utilise technological solutions for health, wellbeing, and community connection. Address barriers to digital access and use, acknowledging the increasing digitalisation of essential services and potential for digital exclusion.
7. **Collaborative Problem-Solving**: Work with service providers to find creative solutions for individual cases and advocate for exceptions or priority access when necessary and appropriate
8. **Critical Analysis and Innovation**: Analyse complex social and health issues, identifying root causes and potential solutions. Apply creative thinking to help individuals and communities reframe challenges and develop innovative approaches to resource utilisation
9. **Enterprising and Adaptive Practice:** Link workers creatively utilize available resources and adapt approaches to local contexts and constraints. They demonstrate resilience and innovation in resource-limited settings, identify opportunities for service enhancement, and develop sustainable solutions using existing community assets. They maintain effectiveness while working within varying resource environments and organizational contexts.

### 4. Community Development and Social Capital Building

*Social prescribing link workers champion community-led initiatives, applying specific skills to catalyse community engagement, build robust social connections, and facilitate initiatives that significantly enhance collective wellbeing and resilience.*

Associated Skills:

1. **Community Engagement and Empowerment**: Actively foster community participation and decisively empower local residents to take leadership roles in community initiatives. Identify and co-create opportunities that deeply resonate with individuals' interests and aspirations, significantly enhancing their skills, knowledge, and confidence to drive effective community action.
2. **Social Network and Capital Building:** Create and strengthen networks of mutual support within the community, facilitating connections between individuals and groups, promoting community resilience through these connections. Foster environments that promote positive relationships, shared experiences, and collective accomplishments.
3. **Inclusive Practice and Cross-Cultural Bridging:** Develop and implement strategies to ensure meaningful participation of all community members, regardless of their background or abilities. Facilitate understanding and cooperation between different cultural, ethnic, or social groups, creating inclusive spaces that respect and affirm diverse identities.
4. **Supporting Community-Led Initiatives**: Proactively support community groups in initiating, planning, and implementing transformative projects that address local needs and enhance community wellbeing. Ensure initiatives are fundamentally community-owned and sustainably led, positioning the community at the forefront of positive change
5. **Collaborative Partnership Development**: Purposefully foster and maintain robust, community-centred partnerships among diverse stakeholders. Skilfully negotiate and manage relationships to achieve shared health and wellbeing objectives, always prioritising community voices and leadership in these collaborations
6. **Navigating Complex Situations:** Link workers understand how to navigate challenging interpersonal and systemic situations while maintaining clear role boundaries. They employ basic de-escalation techniques when appropriate, recognize when to seek support or refer to qualified services, and follow organizational procedures for addressing conflicts. They contribute to positive relationships while clearly understanding the limits of their role in resolving complex situations.
7. **Sustainable Community Capacity Building**: Strategically provide resources and targeted support to enable robust, community-driven project management and long-term community resilience. Actively cultivate and nurture local leadership, fostering the development of self-sustaining community structures that can independently drive ongoing positive change
8. **Community-Led Framework Implementation**: Consistently apply and advocate for a community-led framework in all aspects of social prescribing. Recognise and amplify the community's pivotal role in defining, enhancing, and sustaining their own wellbeing. Actively shift decision-making power to community members, ensuring their voices and experiences drive the direction of initiatives

### 5. Culturally Safe and Inclusive Practice

*Social prescribing link workers demonstrate expertise in providing culturally safe and inclusive services, ensuring that all individuals, regardless of their cultural background, age, or specific needs, can effectively engage with and benefit from social prescribing.*

Associated Skills:

1. **Cultural Competence Development**: Continuously expand knowledge about various cultural groups within the community and regularly reflect on and improve cultural safety in social prescribing practice.
2. **Cultural Safety Implementation**: Apply principles of cultural safety in all interactions, including competence in culturally safe practices for Aboriginal and Torres Strait Islander people. Demonstrate understanding of the historical and ongoing impacts of colonisation on Indigenous communities and create respectful and affirming spaces that recognise the importance of cultural practices, family structures, and community connections
3. **Inclusive Service Delivery**: Ensure social prescribing activities and programs are accessible and beneficial to all community members, with particular consideration for marginalised or underrepresented groups and adapt practices to accommodate diverse beliefs, histories, traits, and experiences related to culture, ethnicity, race, disability, age, gender, sexuality, socioeconomic status, and religion ensuring equitable access and benefits for all community members
4. **Digital Literacy and Inclusive Engagement:** Link workers effectively use digital tools while ensuring inclusive practice for those with varying technological access and comfort. They adapt communication methods for different needs, maintain alternative engagement pathways, and support digital inclusion while recognizing and addressing technological barriers. They leverage technology appropriately to enhance service delivery and reach underserved populations.
5. **Transcultural Connectivity and Adaptability:** Identify and leverage common interests and shared human experiences to foster connections that transcend cultural boundaries. Demonstrate adaptability in facilitating social prescribing activities that unite diverse groups through universal themes of wellbeing, emphasising our shared humanity while respecting individual cultural identities

### 6. Safe and Effective Practice

*Social prescribing link workers demonstrate expertise in identifying, assessing, and mitigating risks to ensure the safety and wellbeing of participants in social prescribing activities. They maintain ethical standards, adhere to legal requirements, and respond effectively to crisis situations, ensuring all practices are both safe and compliant*

Associated Skills:

1. **Comprehensive Risk Management:** Identify, evaluate, and mitigate potential risks in social prescribing activities, including home visits and community-based activities. Implement and maintain appropriate safety measures, guidelines, and procedures. Ensure robust safeguarding practices and confidentiality protocols are in place and regularly reviewed
2. **Navigation of Complex Situations:** Identify signs of complex issues, including potential mental health concerns, domestic violence, and elder abuse. Demonstrate knowledge of appropriate referral pathways and reporting procedures. Apply professional judgement to facilitate timely connections to specialised support services while maintaining clear boundaries of the link worker role.
3. **Ethical Decision-Making**: Apply ethical frameworks to resolve complex dilemmas in social prescribing contexts. Balance risk management with respect for individual autonomy and dignity, considering multiple stakeholder perspectives
4. **Legal Compliance and Documentation**: Demonstrate understanding of legal requirements related to social prescribing practice, including proper note-taking and information disclosure. Maintain accurate, legally compliant records of client interactions, ensuring appropriate consent for information sharing and adhering to privacy legislation
5. **Managing Crises**: Develop and implement emergency response strategies tailored to social prescribing settings and demonstrate readiness to handle emergencies (both medical and non-medical), significant events and natural disasters. Show competence in responding to and managing emergency and crisis situations, employing appropriate interventions and referral pathways as needed.
6. **Adaptive Practice and Safety Enhancement:** Demonstrate flexibility in adjusting risk management and safety practices based on experiences, incidents, and feedback. Provide appropriate support and facilitate reflective learning following challenging events. Regularly assess and update safety protocols, incorporating lessons learned and staying informed about evolving best practices in safe social prescribing. Contribute to the development of improved safety measures that enhance client and practitioner wellbeing.
7. **Conflict Management**: Demonstrate proficiency in identifying, managing, and resolving conflicts at individual, group, and community levels. Apply mediation skills and de-escalation techniques to prevent conflicts from escalating into crises, ensuring diverse voices are heard and respected. Manage and resolve conflicts between individuals, community groups, and local organisations effectively.
8. **Scope Recognition and Referral Navigation**: Link workers understand the boundaries of social prescribing and recognize when clients need additional or alternative support. They maintain knowledge of appropriate referral pathways, particularly for clinical services, and can effectively communicate these boundaries to clients while maintaining supportive relationships. They work collaboratively with other services to enhance client wellbeing within clear professional parameters.

### 7. Data Management and Evaluation

*Social prescribing link workers demonstrate expertise in managing data securely and ethically, while effectively evaluating and reporting on the impact of social prescribing initiatives to drive continuous improvement and advocacy.*

Associated Skills:

1. **Ethical and Legal Data Management**: Maintain accurate, legally compliant records of interactions and outcomes. Implement secure data practices adhering to privacy laws and organisational policies. Understand and apply legal requirements for data collection, storage, sharing, and consent procedures. Regularly update knowledge of relevant legislation and apply compliance measures in daily practice
2. **Evidence Collection for Systems Impact**: Link workers systematically document client journeys, experiences, and outcomes using standardized tools and approaches. They capture patterns in social and practical needs, record successful interventions and challenges, and gather client feedback about service experiences. They contribute valuable ground-level insights about service gaps and solutions while maintaining accurate records of community needs and responses
3. **Feedback Integration**: Implement systems for gathering feedback from service users and partners and integrate feedback into service improvement strategies and program development
4. **Reflective Practice and Continuous Improvement:** Engage in ongoing self-assessment and learning from experiences. Use evaluation findings and reflective insights to inform future planning and enhance personal competencies and service delivery

### 8. Supportive Partnerships and System Change

*Social prescribing link workers skilfully advocate for individuals and systemic change within health and social care contexts. They address barriers to wellbeing, engage in policy discussions, and build partnerships to enhance social prescribing frameworks and broader health policies.*

Associated Skills:

1. **Supportive Partnerships and Advocacy:** Work with individuals to ensure access to services, liaise with providers to accommodate needs, and support applications for services. Identify and address barriers to service access, including informational, physical, transport, financial, and language obstacles.
2. **Aggregating Stories and Data to Identify Systemic Issues:** Identify themes and trends from individual stories and collected data that indicate systemic issues. Analyse and document systemic barriers affecting communities and their environments, considering social, economic, and environmental factors.
3. **Cross-Sector Collaboration:** Develop and maintain partnerships across health, social care, community, and environmental sectors to enhance advocacy efforts and drive collaborative change. Create comprehensive support networks and foster collaborative efforts towards systemic improvements.
4. **Awareness and Communication of Policy Impacts:** Link workers stay informed about how policies and funding mechanisms affect client access and outcomes in their community. They identify and document barriers, enablers, and patterns observed in their work, passing on these insights to supervisors or relevant stakeholders to support service improvements and planning.
5. **Local Needs Assessment and Reporting:** Collect and synthesise local needs data and create reports that can be used to inform larger government planning and commissioning structures (e.g., Regional Needs Assessment).
6. **Systemic Understanding and Contribution:** Demonstrate knowledge of broader health and social care systems and their impact on community wellbeing. Identify opportunities for improvement within scope of practice and contribute to positive change through collaborative efforts and evidence-based insights.

### 9. Integration of Environmental & Contextual Factors in Practice

*Social prescribing link workers demonstrate expertise in incorporating environmental and contextual considerations into their practice, recognising the interconnectedness between individual wellbeing, community health, and environmental sustainability.*

Associated Skills:

1. **Environmental Health and Wellbeing Assessment:** Evaluate how a client's physical and social environment affects their health and wellbeing. Assess the impact of environmental factors on a client's ability to engage in recommended activities. Recognise and document environmental barriers to health and wellbeing at individual and community levels.
2. **Accessibility and Environmental Analysis**: Assess the accessibility of local spaces and activities, considering factors such as mobility, sensory needs, and transportation options. Identify and promote the use of local green spaces for health and wellbeing activities, considering their accessibility and potential health benefits.
3. **Natural Resource Integration and Sustainable Practice:** Incorporate natural environments into social prescribing practices, recognising their role in overall health and wellbeing. Develop social prescriptions that utilise local environmental assets sustainably and promote environmentally friendly community engagement. Integrate respect for the natural world while learning from Indigenous connections to Country.
4. **Environmental Health Awareness**: Link workers understand how environmental factors affect community wellbeing and can connect clients with relevant local environmental programs and green spaces. They maintain awareness of environmental health impacts while working within their scope to support access to environmentally supportive activities and resources.
