# Supplementary material for: Educational standards for Australian social prescribing link workers: a modified Delphi study
Source: Front Public Health. 2026 Feb 19;14:1754668. doi: 10.3389/fpubh.2026.1754668 (PMC12960570; doi:10.3389/fpubh.2026.1754668)
Supplement: Supplementary file 3 [file Table_2.docx]

**Wording may be altered slightly to contextualise the email for specific eligible sub-groups (i.e.. link workers compared to health professionals) and for specific platforms (i.e.. email vs social media).*

**Subject:** Have your say: Social Prescribing Link Work Education Standards in Australia

To [insert name],

ASPIRE (The Australian Social Prescribing Institute of Research and Education) invites you to participate in a Delphi study to review and refine our preliminary Social Prescribing Link Worker Education Standards for Australia. You have been selected for this study based on your expertise and experience in social prescribing.

A Delphi study is a structured communication technique to gather expert opinions and achieve consensus on a specific topic. The process will involve several rounds conducted over several weeks until consensus is reached, with each survey taking approximately 15 minutes to complete.

This process will be conducted online and is best carried out using a desktop or laptop computer, rather than a mobile phone.

If you are interested in participating in the study, please **click here**. For further details, please refer to the attached Explanatory Statement. Your involvement in this study is completely voluntary.

If you know others with social prescribing expertise who may be interested in participating, please forward their contact details to researcher Dr Alessandra Teunisse.

For any questions or queries regarding the project, please contact researcher [insert researcher details]

Kind Regards,

[Lead Researcher details]
